# Mechanistic insights into the aggregation pathway of the patient-derived immunoglobulin light chain variable domain protein FOR005

**DOI:** 10.1038/s41467-023-39280-0

**Published:** 2023-06-23

**Authors:** Tejaswini Pradhan, Riddhiman Sarkar, Kevin M. Meighen-Berger, Matthias J. Feige, Martin Zacharias, Bernd Reif

**Affiliations:** 1 Bavarian NMR Center (BNMRZ), Department of Bioscience, TUM School of Natural Sciences, Technical University Munich, Lichtenbergstr. 4, 85747 Garching, Germany; 2 Institute of Structural Biology (STB), Helmholtz-Zentrum München (HMGU), Ingolstädter Landstr. 1, 85764 Neuherberg, Germany; 3 Center for Functional Protein Assemblies (CPA), Department of Bioscience, TUM School of Natural Sciences, Technical University Munich, Ernst-Otto-Fischer-Straße 8, 85748 Garching, Germany

**Keywords:** Solution-state NMR, Biophysics, Protein aggregation

## Abstract

Systemic antibody light chain (AL) amyloidosis is characterized by deposition of amyloid fibrils. Prior to fibril formation, soluble oligomeric AL protein has a direct cytotoxic effect on cardiomyocytes. We focus on the patient derived λ-III AL variable domain FOR005 which is mutated at five positions with respect to the closest germline protein. Using solution-state NMR spectroscopy, we follow the individual steps involved in protein misfolding from the native to the amyloid fibril state. Unfavorable mutations in the complementary determining regions introduce a strain in the native protein structure which yields partial unfolding. Driven by electrostatic interactions, the protein converts into a high molecular weight, oligomeric, molten globule. The high local concentration of aggregation prone regions in the oligomer finally catalyzes the conversion into fibrils. The topology is determined by balanced electrostatic interactions in the fibril core implying a 180° rotational switch of the beta-sheets around the conserved disulfide bond.

## Abstract (web summary)

Using solution-state NMR spectroscopy, the authors followed the individual steps involved in protein misfolding from the native to the amyloid fibril state for the antibody light chain (AL) amyloidosis related protein FOR005.

## Introduction

Immunoglobulin light chain (AL) amyloidosis is the most common form of systemic amyloidosis^1^. The disease is characterized by the overproduction of an immunoglobulin light chain due to abnormal proliferation of monoclonal plasma cells^2^. The excess light chains are secreted into the plasma and deposit as insoluble amyloid aggregates in the extracellular space of various organs, leading to impaired organ function and failure. Heart involvement is a major risk factor of mortality^3,4^.

Independent of fibril deposition, it has been shown that soluble oligomeric protein has a direct cytotoxic effect on cardiomyocytes. In an isolated mouse heart model, infusion of light chain protein from patients with AL amyloidosis results in diastolic dysfunction similar to that observed in AL amyloidosis patients with cardiac involvement^5^. Amyloidogenic light chain protein thus seems to impair ventricular relaxation, while the contractile function of the heart is preserved. Furthermore, it has been shown that chemotherapy which results in removal of circulating pathogenic light chains yields a drastic reduction of the concentration of biomarkers reporting on cardiac dysfunction^6^. This suggests that cardiac function can be improved despite the fact that the amyloid load in the organ is not altered. It is therefore concluded that soluble immunoglobulin light chain species found in circulation in patients with AL are toxic to cells in target organs^7^.

In contrast to proteins involved in other misfolding diseases such as α-synuclein in Parkinson’s disease or Aβ in Alzheimer’s disease, light chain antibody domains are natively folded once secreted into circulation and have to either partially or fully unfold before they can be converted into amyloid fibrils. It is believed that a decreased thermodynamic stability of the light chain native-state protein is a major factor leading to fibrillation. It was shown that a more highly unfolded intermediate is better suited than a structured intermediate to undergo the topological rearrangements necessary to form amyloid fibrils^8^. The conversion of the natively folded monomer to an amyloid fibril occurs at least via one intermediate state^9^ which is found to be populated in the lag phase of the aggregation process. Intermediates in the aggregation process of light chain antibody domains involve high molecular weight oligomeric structures^10–12^. The intermediate state structures are potential entry point for amyloid aggregation.

The isolated native V_L_ protein undergoes homo-dimerization. The V_L_-V_L_ dimer interface thereby adopts the canonical dimer structure which is also found in the V_L_-V_H_ heterodimer of the antibody native state. In addition, a non-canonical altered dimer structure can be formed in which one monomer is rotated by 90° or 180°^13,14^. The altered dimer structure is considered to be an important factor to promote amyloidogenicity. More recently, Ramirez-Alvarado and co-worker have found a dependence between the altered dimer conformation and the formation of stable oligomeric intermediates^10^. On the other hand, stabilization of the native homo-dimer interface by small molecules allows to prevent unfolding and thus fibril formation^15–17^. The mode of action of these drugs is similar to tafamidis in transthyretin^18^, and thus of high pharmacological interest.

The exact molecular mechanisms of these early events in the aggregation process of immunoglobulin light chains are not well understood. Recently, the fibril structure of the patient derived immunoglobulin light chain protein FOR005 was characterized by MAS solid-state NMR^19,20^ and cryo-EM^21^. We found that the somatic mutation R49 is of particular importance for fibril stability. In this manuscript, we investigate in detail the aggregation kinetics of this protein from the native state to an oligomeric intermediate state using solution-state NMR and other biophysical techniques. We find that the patient protein FOR005 forms several intermediates that have distinct chemical shifts. Fibril formation of FOR005 is prevented at high concentrations which favor the canonical dimeric state. By contrast, the protein coding for the closest germline sequence does not populate any intermediate state. We show that tension in the backbone conformational space of CDR loop residues and electrostatic interactions are the driving force for conversion into a high molecular weight, oligomeric, random coil structure that precedes amyloid formation.

## Results

FOR005 is a patient-derived λ-III LC sequence^20,22^. The respective germline (GL) is mutated at five positions, namely at residues S31Y, F48Y, R49G, S51N and A94G (mutations indicate transitions from patient to the GL protein). The importance of the individual mutants with respect to aggregation propensity and thermodynamic stability has been studied recently^23^. In addition to the patient and the germline sequences FOR005 and GL, we have investigated the single-point mutant FOR005-R49G. To find out whether the patient mutations affect the native immunoglobulin fold, we carried out solution-state NMR assignment experiments. In particular, we have recorded the triple resonance assignment experiments HNCACB, HN(CO)CACB, and HNCA to yield sequential connectivities for FOR005, GL, and the single point mutant FOR005-R49G. We find that chemical shift perturbations induced by the point mutants are localized around the respective site of mutagenesis in all cases (Supplementary Fig. 1). The solution-state and solid-state NMR chemical shifts are deposited in the BMRB under the accession code BMRB-ID 50211 and 50192, respectively.

### Biophysical characterization of FOR005 oligomerization

**Fig. 1:**
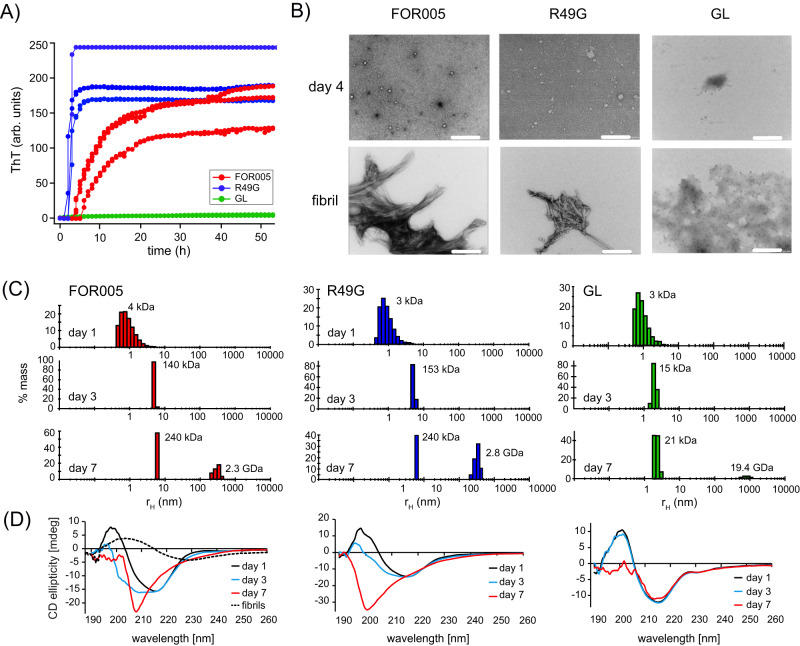
Biophysical characterization of FOR005 oligomers. **A** The patient protein FOR005 (red), the single point mutant R49G (blue) and the germline protein GL (green) were incubated at a concentration of 25 μM and 37 °C. FOR005 and R49G aggregate on a similar time scale, while GL does not aggregate in the time course of the experiment. For each protein, three replicates were recorded. To facilitate aggregation, 500 μM SDS was added to all protein solutions. In the absence of SDS, aggregation is very slow and requires several weeks to yield amyloid fibrils. **B** Representative EM images for FOR005, R49G and GL taken at different days after incubation. The aliquot for the fibril sample was taken after 30 days of incubation. The scale bar represents 200 nm. **C** DLS size distribution of FOR005 (red), R49G (blue) and GL (green) initially (top) and after and incubation time of 3 (middle) and 7 days (bottom). The scattering data is shown in a mass weighted representation. All experiments are carried out using a protein concentration of 50 μM. **D** Far UV CD data for FOR005, R49G and GL recorded as a function of time. The fibril sample (dashed, black line in the left spectrum) was taken after 30 days of incubation and is shown as a reference. A protein concentration of 50 μM protein was employed for all measurements. In each case, the buffer spectrum was subtracted from the protein data. Source data are provided as a Source Data file.

At a concentration of 50 μM, FOR005 converts to amyloid fibrils with a characteristic time on the order of 2–3 weeks (Fig. 1A, B). The mutant R49G aggregates on a similar time scale while GL does not form any ThT positive aggregates at all (Supplementary Fig. 2). Light chain antibody domains involved in AL-amyloidosis are known to populate oligomeric intermediates^10,11^. In order to find out whether these intermediates are as well formed by FOR005, we carried out dynamic light scattering (DLS) experiments (Fig. 1C). Freshly dissolved patient protein yields an average hydrodynamic radius of 1.2 nm which corresponds to a molecular weight of 8 kDa for a globular protein and which fits the FOR005 native state. After an incubation time of 1 week, an average hydrodynamic radius of 5–6 nm is observed. This hydrodynamic radius corresponds to a molecular weight of 140–240 kDa and represents a low molecular weight oligomeric intermediate. The DLS profile yields no residual scattering intensity originating from the monomeric protein. Again, R49G shows a similar behavior as the patient protein, while the scattering profile for GL remains almost unaltered in the time course of 1 week. Of note, the DLS experiments for FOR005 and R49G after 1-week yield scattering intensity from particles with a hydrodynamic radius of ca. 200 nm. The DLS results are in agreement with DIC light microscopic experiments in which we observe high molecular weight oligomeric aggregates (Supplementary Figs. 3, 4). Circular dichroism experiments indicate that FOR005 adopts a random coil conformation in the lag phase of the ThT experiment (Fig. 1D). Similar as for the patient protein, the CD spectra of R49G show a conversion to random coil, while GL retains the native fold.

The FOR005 oligomers are stable against chemical denaturation. In sodium dodecyl-sulfate polyacrylamide gel electrophoresis SDS PAGE (Supplementary Fig. 5), the intensity of the monomer FOR005 band is getting smaller over time, while higher molecular weight bands appear indicating the formation of SDS stable oligomers and aggregates. At the same time, there are no changes observed in the SDS PAGE for GL. The gels suggest that the proteins are not degraded over time. This is confirmed by an ESI-MS analysis which shows that the chemical integrity of all samples (FOR005, R49G, GL) is preserved in the time course of the experiments (Supplementary Fig. 5).

### Characterization of the V_L_ homo-dimer interface

**Fig. 2:**
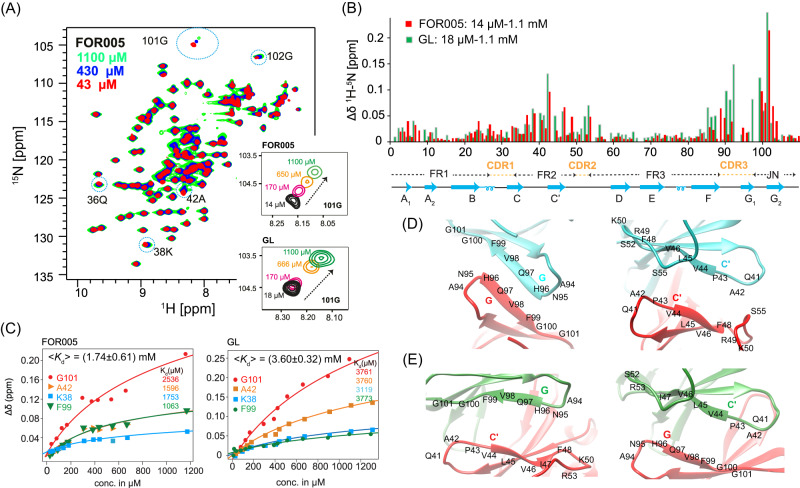
Monomer-dimer equilibrium in FOR005 and GL. **A** Concentration-dependent ^1^H,^15^N HSQC spectra for the patient protein FOR005. Protein concentrations of 14/18 μM (black), 170 μM (red), 650 μM (orange) and 1.1 mM (green) have been employed. Residues that show large concentration dependent chemical changes are highlighted with a dashed blue circle. In the inset, the spectral region containing G101 is shown enlarged for FOR005 and GL. **B** ^1^H,^15^N chemical shift difference Δδ for spectra recorded at the highest and lowest concentration for FOR005 (red) and GL (green) as a function of residue. **C** Protein concentration dependent chemical shift difference Δδ for FOR005 (left) and GL (right). To fit the dissociation constant *K*_d_, the chemical shifts of the residues K38, A42, F99 and G101 were employed. The non-linear fit of the dilution data yields (1.74 ± 0.61) mM and (3.60 ± 0.32) mM for FOR005 and GL, respectively. Structural models of the canonical (**D**) and alternate (**E**) dimer interfaces. The crystal structure of FOR005 VL (PDB 5L6Q) was employed to represent the canonical dimer interface. To obtain the alternate dimer structure a homology model has been generated using the X-ray structure of AL09 H87Y (PDB: 2KQN). Source data are provided as a Source Data file.

First, we asked the question whether V_L_ aggregation is concentration dependent and if high concentrations catalyze the conversion to fibrils. V_L_ proteins are known to have a certain tendency to homo-dimerize^13^. The patient protein FOR005 crystallizes into a canonical dimer^22^. The interaction between two proteins in solution is however rather weak (with a *K*_d_ in the mM range). NMR resonances at the dimer interface are therefore broadened due to chemical exchange^24^. To characterize the dimer properties, we measured concentration dependent ^1^H,^15^N HSQC spectra for both FOR005 and GL (Fig. 2A). Due to chemical exchange broadening, a few residues located in strands C’ and G1 are not observable in both the patient and germline protein. The protein concentration was varied by dilution from 1.1 mM to 14 μM in 13 steps for FOR005, and from 1.3 mM to 18 μM in 14 steps for GL. The chemical shift difference between the spectrum recorded at the lowest and the highest concentration is represented in Fig. 2B. For both proteins, we observe the largest chemical shift changes in strands C’ and G2. To determine the dissociation constant *K*_d_ for dimer formation, we employed the concentration dependent chemical shift for K38, A42, F99 and G101 (Fig. 2C), following the protocol by Ramirez-Alvarado and co-workers^14^ (see Methods also). We obtain a *K*_d_ of (1.74 ± 0.61) mM and (3.60 ± 0.32) mM for FOR005 and GL, respectively. Even though the *K*_d_ value for dimerization is an order of magnitude higher than the concentrations used in the NMR experiments, dimerization can kinetically inhibit aggregation as the monomer-dimer equilibrium is fast in comparison to the rate of unfolding. However, the *K*_d_ value for FOR005 is slightly lower than the respective value for GL, suggesting that homo-dimerization alone cannot explain the increased aggregation propensity of FOR005.

It is known, that the V_L_ dimer interface can adopt different topologies^13,14^. In the canonical dimeric form, strands G1 (N95-F99) as well as strand C’ (Q41-S55) interact and from a G1-G1/C’-C’ interface (Fig. 2D). In the altered dimer structure, one protomer is rotated by 90° with respect to the other protomer^14^. As a consequence, strand G1 packs against residues involving C’. We observe significant differences in the loop region connecting strands F and G1 for FOR005 and GL (Fig. 2B), involving in particular residues Y85-S92. None of the dimer models allow to explain the concentration-dependent chemical shift changes observed for strand F (residues Y85-S92). Similarly, no effects for residues F99-T103 are expected from the dimer topology. Residues located in strand F are actually shielded from the dimer interface and should not at all experience a concentration-dependent chemical shift.

### Aggregation kinetics monitored by solution-state NMR

**Fig. 3:**
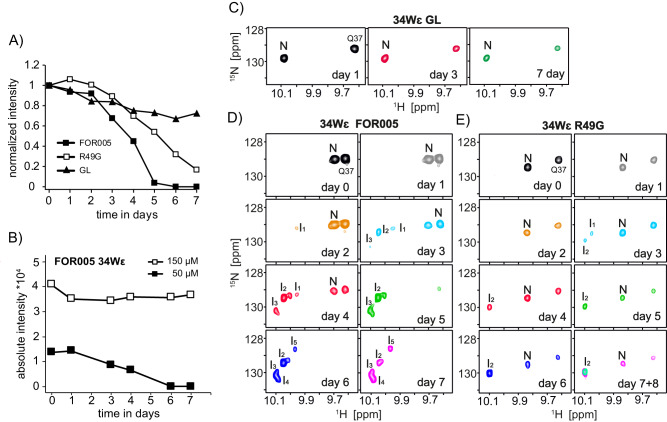
Aggregation kinetics for different FOR005 variants using solution-state NMR. All experiments were performed at a temperature of 25 °C. **A** Bulk NMR signal intensities for the patient protein FOR005 (closed squares), the germline protein (closed triangles) and the single point mutant FOR005-R49G (open squares). In the analysis, the average cross peak intensities of the backbone amides of G15, I57 and E82 were taken into account. **B** Aggregation kinetics of FOR005 at a protein concentration of 50 μM (closed squares) and 150 μM (open squares). For quantification, the cross peak intensity of the side chain cross peak of W34ε has been employed. **C** Tryptophan spectral region of the ^1^H,^15^N HSQC for GL, FOR005 (**D**) and R49G (**E**) as a function of time. In all samples, a protein concentration of 50 μM was employed. N and I refer to the natively folded and intermediate states, respectively. For FOR005 and R49G, N disappears over time, while the cross peak remains invariant in GL. New appearing peaks in FOR005 are labeled as I_1_-I_5_. For R49G, only one predominant intermediate is observed. Source data are provided as a Source Data file.

Next, we analyzed the kinetics of the aggregation process in more detail, with the aim to identify potential intermediate states. All FOR005 constructs employed here contain only a single tryptophan residue and allow to follow its NMR peak intensity over time. We recorded ^1^H,^15^N HSQC experiments for the patient protein FOR005, its germline variant GL and the single point mutant FOR005-R49G over a period of seven days (Fig. 3A). All experiments were performed at 25 °C using the same acquisition parameters and NMR spectrometer. The data confirms that GL is the most stable protein, while FOR005 shows the fastest aggregation kinetics with FOR005-R49G being in-between these two extremes. We observe a significant concentration dependence of the aggregation kinetics (Fig. 3B). While the ^1^H,^15^N HSQC resonances of FOR005 decay fully at a concentration of 50 µM, almost no decay is observed at a concentration of 150 µM within a time period of 7 days. The NMR experiments are in agreement with concentration-dependent ThT experiments (Supplementary Fig. 2) which show that the aggregation lag time for FOR005 has a minimum at a concentration 50 μM. This finding is consistent with earlier reports that have shown that high concentrations and dimerization are protective against protein aggregation and fibril formation in immunoglobulin light chain variable domains^8,25,26^. We would like to note here that it is difficult to yield absolute comparable time scales for the aggregation kinetics or critical concentrations in the different experiments, as aggregation depends on parameters such as the geometry of the sample container affecting the air/water interface. Similarly, the SDS added in the ThT experiments enhances the rate of aggregation significantly.

For GL, only a single W34ε cross peak representing the native state is observed for all time points (Fig. 3C). For the patient protein FOR005, a weak cross peak (I_1_) is detected after 2 days, that increases in intensity until day 3 and then disappears within the next 24 h (Fig. 3D). At the same time, two more intermediates I_2_ and I_3_ are populated that remain until the end of the kinetics. At day 5 and 6, two more conformers I_4_ and I_5_ can be detected. The data suggests that FOR005 fibrils can form via several distinct intermediates. At this point, we cannot differentiate whether these intermediates are oligomers of increasing molecular weight or whether different off-pathway intermediates are populated that decay when the equilibrium is shifted further towards the fibril state.

By contrast, the kinetics for R49G are less complex and show one predominant intermediate state (Fig. 3E). The amide backbone NMR spectra of FOR005, GL, and R49G are shown in Supplementary Fig. 6. For the patient protein, new peaks become visible after 3 days at proton chemical shift around 8.0 ppm indicating unfolding of the protein. After seven days, all native peaks have disappeared. By contrast, no new peaks are visible in the GL spectra after the same time period. During this time, the cross peak intensity decreases only marginally. The single point mutant FOR005-R49G shows an intermediate behavior, with resonances representing a mixture of native and unfolded protein. The kinetics of the tryptophan side chain peak intensities is shown in Supplementary Fig. 7A. Formation of large oligomeric intermediate states is in agreement with Diffusion Ordered Spectroscopy (DOSY) NMR experiments which show that the rate of translational diffusion of the patient protein FOR005 decreases when the protein converts into an unfolded state after an incubation time of 7 days (Supplementary Fig. 7B).

It is known that directly after dissolution micelle like structures or lag-free globular oligomers are formed at the onset of the aggregation kinetics^27,28^. We wanted to find out, whether these early aggregates potentially affect the aggregation behavior. We therefore compared FOR005 patient protein solutions that are filtered using a 0.22 μm cut-off membrane with a FOR005 protein solution that has been sedimented in a ultracentrifuge prior to the experiments. Sedimentation of the protein solution prior to the experiment removes off-pathway aggregates (Supplementary Fig. 12). We show that pre-sedimentation, however, has only a minor effect on the aggregation kinetics and no effect on the intermediates that are formed.

To rule out the possibility that FOR005 intermediates are only populated since the mutant is thermodynamically less stable, we performed NMR experiments at different temperatures. The melting temperature of FOR005 is decreased by 12.8 °C in comparison to its germline variant^23^. The NMR aggregation kinetics for FOR005 and GL (Fig. 3) were recorded at a temperature of 25 °C. These experiments were repeated at a temperature of 15 °C and 37 °C for patient and germline, respectively (Supplementary Fig. 8). We find that both proteins behave exactly the same as at 25 °C, indicating that the protein stability is not sufficient to explain the amyloidogenicity of the patient protein.

### R49G locally unfolds involving CDR3

**Fig. 4:**
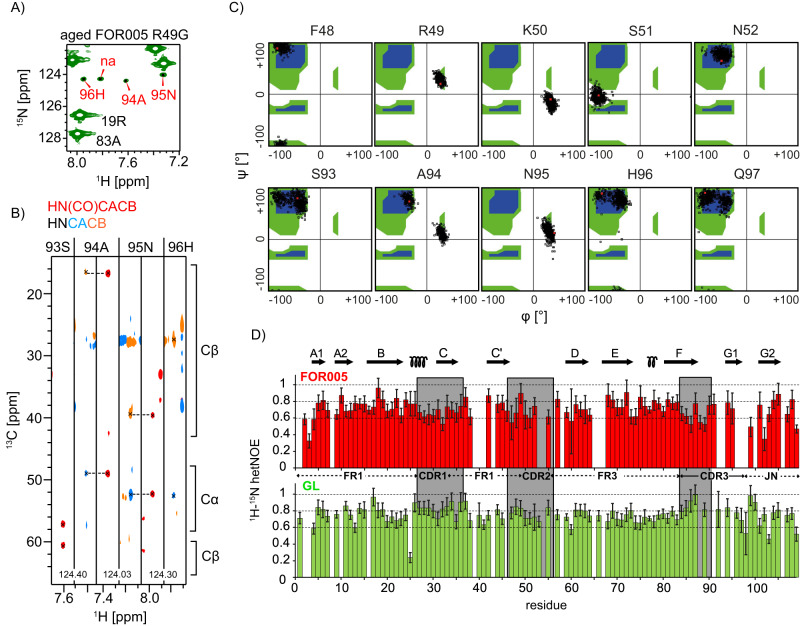
Detection of a partially folded intermediate state for R49G. **A** Selected region of the ^1^H,^15^N HSQC spectrum obtained for a several week old R49G sample. At low contours, additional peaks are observed that can be sequentially assigned to residues located in CDR-3. **B** Sequential walk involving residues S93-H96 of the minor populated state of FOR005-R49G. The assignment is obtained from 3D HNCACB and HN(CO)CACB experiments. **C** Distribution of backbone dihedral angles φ and ψ during the MD simulation of FOR005. The sampled backbone dihedral angles are represented in a Ramachandran diagram for residues 48 to 52, and 93 to 97 (black open squares). The calculations show that backbone conformations in CDR2 and CDR3 are energetically unfavorable. Favorable regions are indicated in blue, the extended allowed region in green. **D** Heteronuclear NOE experimental data for the backbone amides of FOR005 (red) and GL (green). The experiments were carried out at a magnetic field strength of 11.75 T, corresponding to a ^1^H Larmor frequency of 500 MHz. The temperature was set to 25 °C. In both samples, a protein concentration of 50 μM was employed. Error bars were extracted from the signal-to-noise ratio of the experiment recorded with and without proton decoupling prior to acquisition of ^15^N.

For R49G, the tryptophan resonance reflecting the native state can be tracked until the end of the experiment after 7 days. This single mutant FOR005-R49G apparently populates a predominant oligomeric intermediate. This opens the possibility to identify regions in the protein which participate in early unfolding events. Figure 4A shows a selected region of the ^1^H,^15^N HSQC spectrum obtained for FOR005-R49G obtained from a sample that was aged for several weeks. The full spectrum is shown in Supplementary Fig. 9. At low contours, additional peaks are observed that can be sequentially assigned to the loop CDR-3 (S93-H96) (Fig. 4B). To identify dynamic regions in FOR005, we performed hetNOE experiments (Fig. 4D). Large hetNOE values indicate rigid structures, while lower hetNOEs are due to faster time scale motional processes which would be expected for non-structured loops and termini. Surprisingly, we find that all CDR loops as well as the termini adopt rather large hetNOE values. This is consistent with the observation that the N- and C-termini are buried in the core. Loops are not flexible but are stabilized by many hydrogen bonds and hydrophobic interactions. Comparing hetNOE data of FOR005 and GL, we find that residues 27–36 and 84–89 yield smaller hetNOE values in the patient sequence, suggesting that the patient mutations induce a subtle destabilization of the CDR-1 and CDR-3 loops. This finding is in agreement with molecular dynamics simulations which suggest that Ala-94 in CDR-3 in FOR005 induces a greater loop dynamics inducing exchange between two backbone conformations (Fig. 4C). In fact, the backbone torsional angles in the loops CDR-2 and CDR-3 populate unfavorable regions in the Ramachandran space, suggesting that the proteins of these particular patient variants have a higher probability to unfold. For comparison we also performed simulations using the germline protein GL (Fig. S10). Indeed, the substitutions R49G and A94G in the germline sequence relax the sterical strain, as glycine residues readily adopt conformations with positive backbone phi values. Note that the stabilization induced by the glycine substitutions is also reflected in the significantly higher melting temperatures of the germline with respect to the patient protein^23^. In addition, the substitutions R49G and A94G allow to relax sterical strain in neighboring residues imposed by the fold. E.g. K50 partially samples favorable α-helical conformations in GL. At the same time, additional alternative CDR-3 loop conformations are sampled for GL.

### Assignment of dynamic residues in the molten globule oligomeric state of FOR005

**Fig. 5:**
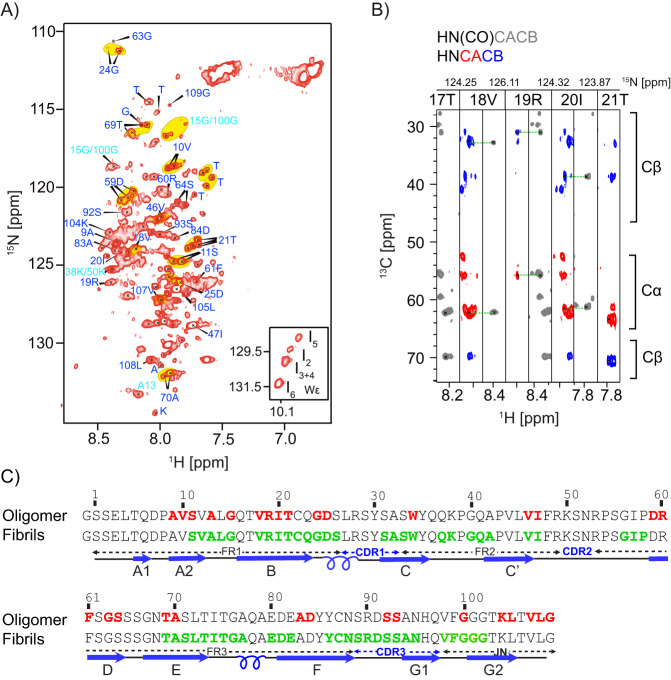
Non-structured residues in oligomeric FOR005. **A** Assigned ^1^H, ^15^N HSQC spectrum of an aged FOR005 sample. Peaks highlighted in yellow belong to the same spin system. Tryptophan resonances are shown in the inset. **B** HNCACB/HN(CO)CACB strip plot involving residues P8-S11 and T17-T21 with sequential assignments. Above pairs of strips, the ^15^N chemical shift of the connecting amide is indicated. **C** Assigned residues in the FOR005 fibrils and the oligomeric intermediate state are indicated in green and red, respectively. The assigned amyloid core residues in the fibril state are taken from Pradhan et al.^19^. Secondary chemical shifts for all assigned residues in the oligomeric-state are random-coil like (data not shown). The secondary structure elements of the native state are shown on the bottom of the figure to guide the eye.

Next, we were aiming for the NMR spectroscopic analysis of the FOR005 oligomer. The experimental spectra are characterized by peaks that reflect disordered residues. Our strategy was to assign the NMR chemical shifts of residues in mobile regions and to identify the residues that stabilize the oligomer by complementarity. For this purpose, we employed a 1 mM FOR005 patient protein sample that was allowed to age for 1 month and recorded backbone assignment experiments. The high concentration ensures that protein aggregation is slowed down which facilitates the assignment process. In particular, we have recorded 3D HNCACB and HN(CO)CACB experiments to unambiguously identify all amino acids types. The fact that more than one intermediate is observed for most residues complicated the analysis. Nevertheless, we could assign 29 residues for the oligomeric intermediate state (Fig. 5A). Strip plots involving residues T17–T21 are shown in Fig. 5B. The secondary chemical shift analysis for the assigned residues shows that all residues adopt a random coil like conformation (data not shown), suggesting that these residues have very high mobility and are not part of the core structure of the oligomeric intermediate. This is consistent with the CD spectra (Fig. 1) which show that FOR005 adopts a mostly random-coil structure in the lag phase before amyloid fibrils are formed. In turn, this implies that non-assigned residues should be part of the core of the intermediate structure and involved in intermolecular interactions. The assigned residues of a FOR005 oligomer and a fibril sample are compared in Fig. 5C. The amyloid core region is taken from Pradhan et al.^19,20^. The largest consecutive stretch of residues that is not assigned involves residues S26-L45, F48-P58, and S71-E82 (strands B-CDR1-C, CDR-2 and E).

### Ionic strength dependence of the FOR005 oligomeric assembly

**Fig. 6:**
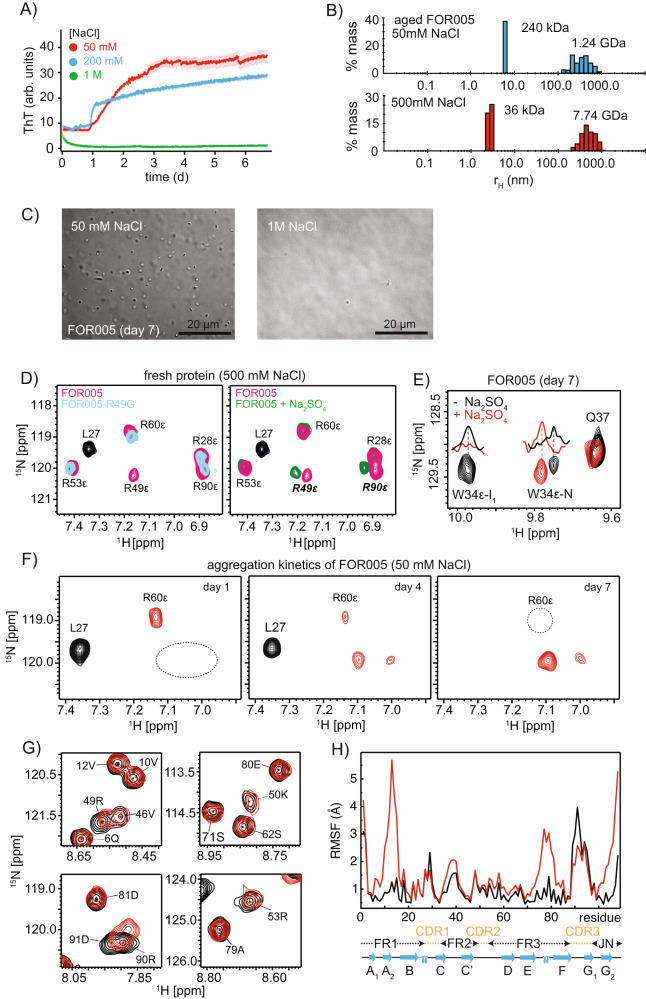
Salt shields electrostatic interactions and prevents formation of oligomeric FOR005. **A** ThT aggregation assay for a 50 µM FOR005 solution as a function of the NaCl concentration. Curves represented in red, blue and green are obtained from preparations containing 50 mM, 200 mM and 1 M NaCl. To facilitate aggregation, 500 μM SDS was added to all protein solutions. *n* = 3 independent ThT experiments for three different salt concentrations have been recorded. For 50 mM, 200 mM and 1 M NaCl, a lag time of (0.70 ± 0.38)d, (1.73 ± 0.32)d and ∞ has been observed. **B** DLS data for a 7 days old sample in presence of 50 mM (blue) and 500 mM NaCl (orange). Addition of salt dissolves low molecular weight oligomers (particles with hydrodynamic radius of 8 nm) and recovers the characteristic scattering for the monomeric protein. The sample was not pre-sedimented prior to the start of the kinetics. **C** Representative Differential Interference Contrast (DIC) microscopy images of the patient protein FOR005 in the presence of 50 mM NaCl (left) and 1 M NaCl (right). The FOR005 sample was pre-sedimented before the start of the kinetics and then incubated for 7 days. Addition of salt induces the disappearance of high molecular weight aggregates. The scale bar corresponds to a length of 20 μm. **D** Solution-state ^1^H,^15^N HSQC spectra with focus on the arginine-ε spectral region. (Left) comparison of spectra obtained for fresh samples of FOR005 (red) and FOR005-R49G (blue). The side chain resonance of R49 is readily assigned. The experiments were recorded in the presence of 500 mM NaCl to reduce the hydrogen exchange dynamics. (Right) spectra recorded in the absence (red) and presence (green) of 100 mM sodium sulfate. R49ε and R90ε show significant chemical shift perturbation suggesting that these arginines are susceptible to a negatively charged environment. The arginine side chain resonances are folded into the amide spectral region and yield negative cross peak intensity (red/blue/green) with respect to the amide backbone resonances (L27, black). **E** Superposition of ^1^H,^15^N HSQC spectra obtained for an aged 50 μM sample of the patient protein FOR005 in the presence (red) and absence of 100 mM sodium sulfate (black). Addition of sulfate recovers the native state. **F** NMR kinetics of the FOR005 patient protein. The spectra show the folded arginine side chain resonances in red. The side chain resonance of R60 is protected against exchange in the native state due to its involvement in a salt bridge with E80/D81. During aggregation, the resonances of R60-ε as well as the resonances of the native state (L27, in black) disappear. At the same time, two side chain resonances appear which originate from exchange protected arginines in the unfolded state. **G** Titration of the FOR005 derived peptide 79-AEDEADYY-86. The figure shows a superposition of a selected region of the ^1^H,^15^N HSQC spectra of FOR005 recorded in absence (black) and presence of a 10x molar excess of the 8-residue peptide 79-AEDEADYY-86 (red). Amide resonances of positively charged side chains such as R49, K50, R53 and R90 show significant chemical shift perturbations upon titration. **H** MD simulations of FOR005 and FOR005-R60A. The root-mean-square fluctuations (RMSF) is represented as a function of residue for the MD trajectory (1.6 μs, at 310 K) for the patient sequence FOR005 (black line) and the single point mutant FOR005-R60A (red line). Source data are provided as a Source Data file.

We speculate that electrostatic interactions drive the oligomeric assembly, given the fact that R49 is an important mutation that has a dramatic effect on the thermodynamic properties of the native state as well as on the aggregation kinetics of the patient protein. Recent evidence shows that oppositely charged intrinsically disordered proteins (IDPs) can form high-affinity complexes that involve neither the formation of secondary or tertiary structure nor site-specific interactions between individual residues^29^. Phase separation in case of IDPs is often induced by formation of coacervates from positively and negatively charged polyelectrolyte IDPs^30,31^. In order to find out whether the oligomer assembly is in fact driven by electrostatic interactions, we performed aggregation experiments under conditions of high salt. In presence of 1 M NaCl the light chain protein does not form fibrils anymore in ThT experiments (Fig. 6A). This is in agreement with DLS experiments (Fig. 6B) which show that the 12 nm scattering peak observed for a 7 days old FOR005 sample is shifted to a hydrodynamic radius of 5 nm, after salt is added to the protein solution. This suggests that NaCl either stabilizes the native state of the protein or shields charged side chains to prevent intermolecular electrostatic interactions. The DLS data are in agreement with DIC experiments which show that high concentrations of salt dissolves the high molecular weight oligomeric aggregates (Fig. 6C). To differentiate between a stabilization and shielding effect, we probed the susceptibility of the arginine side chains to negative charges (Fig. 6D). Charged side chains in the micro-environment of an arginine side chain, but also salt have an impact on the hydrogen exchange rates of arginine side chain exchangeable groups^32–34^. We therefore added 500 mM NaCl to freshly dissolved protein to facilitate the observation of arginine-ε protons. The side chain resonance of R49 is readily assigned by comparing the spectra of FOR005 and FOR005-R49G. Addition of sulfate results in a strong chemical shift perturbation of the resonances R49-ε and R90-ε suggesting that these two side chains are susceptible to negative charges. Further, we recorded solution-state NMR spectra for an aged FOR005 sample to find out whether sulfate is able to shield electrostatic interactions and prevent formation of oligomeric intermediate states. The W34 tryptophan side chain HSQC spectra of the aged reference sample are represented in black, while the experimental spectrum after titration with sulfate is shown in red (Fig. 6E). We observe that addition of sulfate recovers the native state. At the same time, the intermediate W34-ε peak disappears quantitatively upon titration with sulfate, suggesting that electrostatic interactions are indeed crucial to drive formation of the oligomeric assembly. To obtain a direct hint that electrostatic interactions stabilize the oligomeric state, we monitored the arginine side chain region of the HSQC spectrum over a period 1 week (Fig. 6F). While resonances reflecting the native state disappear over time, new arginine side chain resonances are observed after 4 days indicating that some arginine side chains are protected in the unfolded state. This suggests that electrostatic interactions in fact stabilize the oligomeric state.

### Destabilization of the FOR005 native state by competition for the conserved R60-D81/K39-E80 interactions

Next, we wanted to identify the residues which are the target of the exchange-protected arginines in the intermediate state. We speculate that the conserved negatively charged patch containing residues E80/D81/E82 is the target of the positively charged arginines. We therefore titrated the FOR005-derived peptide AEDEADYY (residues 79–86) to the protein sample (Fig. 6G). We observe significant chemical shift changes for R49, K50, R53 and R90, suggesting that these residues are susceptible to negative charges, and can potentially recognize the conserved negatively charged region of the protein.

To further validate the hypothesis that competition for electrostatic interactions is the driving force for the unfolding of the native state protein, we performed MD simulations (Fig. 6H). We assume that competition for the salt bridge R60-D80/E81 by R49 results in a destabilization of the protein. In fact, it has been shown experimentally that the mutation R60A has a dramatic impact on the fibril formation kinetics and the thermodynamic stability of the V_L_ domain^35^. For the patient protein FOR005, high RMSF fluctuations are found in particular for CDR-3 (residues 89–95). At the same time, we observe that backbone dihedral angles can adopt unfavorable values in this part of the protein (Supplementary Fig. 10). To study the influence of the salt bridge R60-D80/E81 on protein stability, we repeated the MD simulations for the single point mutant R60A. We observe a further destabilization of the protein and increased RSMF fluctuations for the loops connecting strands A2-B, E-F, as well as for the N- and C-terminus (Fig. 6H), suggesting that the concerted action of loop instabilities in CDR2 and weakening of the salt bridge R60-D80/E81 by electrostatic competition results in faster aggregation and fibril formation.

## Discussion

**Fig. 7:**
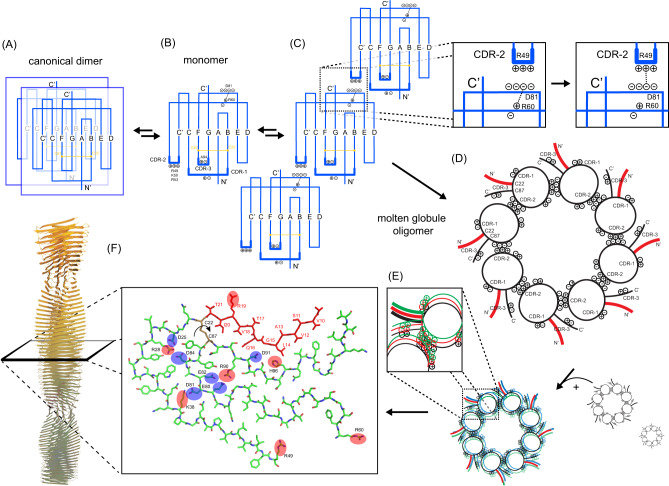
Postulated aggregation pathway of the antibody light chain VL domain FOR005. **A** Dissociation of the homodimer is necessary to initiate aggregation. Even though patient and germline protein have comparable affinities, dimerization in the patient protein is perturbed by increased dynamics in CDR-3 induced by the single point mutation G94A (**B**). **C** Electrostatic interactions between the positively charged CDR-2 and the negatively charged conserved patch E80-D81-E82 drives intermolecular interactions. Destabilization of the salt bridge R60-E80/D81 by competition with CDR-2 yields unfolding of the VL domain and induces an oligomeric molten globule state (**D**). The loop induced by the disulfide bridge between C22 and C87 adopts a random-coil like conformation. The aggregation prone N-terminal region is color coded in red. **E** Subsequent addition of multiple molten globule oligomers results in a high molecular weight assembly in which the aggregation prone N-terminal region is aligned in parallel and in-register. **F** Conversion into the fibrillar state by transition from side chain-side chain to backbone-backbone hydrogen bonding. The topology is adopted from the cryo-EM structure of ex vivo FOR-005 fibrils (Radamaker et al.^21^) (PDB ID: 6Z1O), and is found as well in vitro in seeded preparations. Positively and negatively charged side chains are highlighted in red and blue, respectively. MD simulations suggest that only one orientation of the large loop induced by the disulfide bridge between C22 and C87 is energetically favorable.

Our data allows to draw a picture of the aggregation pathway from the native homo-dimeric structure to the fibrillar structure in the aggregated state (Fig. 7). We find that only the monomeric V_L_ protein is capable to form fibrils, while the homodimeric protein is protected against aggregation (Fig. 7B). We can exclude that misfolding involves population of an alternative dimer interface. FOR005 has to quantitatively unfold and oligomerize before fibril formation sets in. We find that the CDR-3 loop residues adopt either unfavorable backbone torsional angles or are in exchange between different conformational states. As CDR-3 is part of the dimerization interface, CDR loop dynamics thus has consequences for dimerization. This is also reflected in differences in the concentration-dependent chemical shift changes between patient and germline protein.

The mutation G94A destabilizes the CDR-3 loop and induces a partial unfolding of strand F. Increased loop dynamics is confirmed by the hetNOE data which shows decreased values for CDR-1 (residues 27–36) and CDR-3 (residues 84–89) in the patient protein. We thus suggest that patient mutations in the CDR loops CDR-2 and CDR-3 induce a destabilization of the native fold which facilitates unfolding. An early local unfolding event is observed for the single point mutant FOR005-R49G for which we observe a second set of peaks for residues located in strand F and CDR-3.

The thermodynamic analysis of FOR005 has shown that CDR-2 and the mutation G49R (from germline to patient) play an important role for protein aggregation and fibril formation. In the patient protein, R49 is solvent exposed and cannot contribute to a destabilization of the protein. We therefore suggest that intermolecular interactions stabilize a transition state structure which would explain the reduced stability of the patient mutant. In the patient protein FOR005, CDR-2 is highly positively charged and can potentially interact with an oppositely charged region of the protein. Such a region is found in the conserved cluster of negative charges E80-D81-E82 in the loop connecting strands E and F. Competing electrostatic interactions weaken the conserved hydrogen bonding network involving K38-E80 and R60-D81, and result in protein unfolding. In fact, it is known that the salt bridge R60-D81 is crucial for protein stability. The mutated V_L_ protein R60A has a dramatically reduced stability and readily forms fibrils^35,36^. This is reflected as well in our MD simulations (Supplementary Figs. 10, 11). The mutation R60A results in increased dynamics in the N- and C-termini, as well as in the loops connecting the strands A2-B and E-F’.

The mutation G49R and G94A thus exert a concerted effect with respect to aggregation. While G94A yields a local destabilization of CDR-3 and unfolding of strand F, G49R competes for the conserved salt bridge R60-D81 and induces simultaneously unfolding and oligomer formation.

We show that electrostatic interactions stabilize the oligomer: Conditions of high salt abolish fibril formation (Fig. 6A) and allow to shift the misfolding equilibrium back towards the native state (Fig. 6B, E). In the native state and at low salt concentration, only the side chain resonance R60-ε is visible as it is involved in a salt bridge to E80/D81. During aggregation, the side chain resonance of R60 disappears (Fig. 6F). At the same time, two new protected arginine side chain resonances are observed. This is remarkable given the fact that the protein is unfolded at this stage. Arginine side chain resonances can be only observed if they are involved in electrostatic interactions in the unfolded state. We therefore assign these two arginine side chain resonances to R49 and R90, since both side chains are susceptible to negative charges (Fig. 6D). In the native state structure, both side chains are close in space, and point into the same direction. We speculate that R49 and R90 attack the salt bridges K38-E80 and R60-D81, unfold the patient protein and form an alternative electrostatic network.

The involvement of two interfaces—a positively charged interface in CDR-2 and a negatively charged region in the loop connecting strands E and F—allows to build large oligomeric complexes (Fig. 7D). The data that we present are in agreement with IDPs that employ charge–charge interaction to undergo liquid–liquid phase separation (LLPS)^29–31^. Intrinsically disordered proteins (IDPs) are characterized by a lack of defined structure. Instead, they populate ensembles of rapidly interconverting conformations with marginal structural stabilities. Changes in solution conditions such as temperature and crowding agents consequently affect IDPs more than their folded counterparts. The residual structure content of IDPs is modulated both by ionic strength and by the type of ions present in solution. Similarly, the affinity of IDPs to their targets is modulated by ion-specific changes in kinetics and residual structure^37^. In this sense, the misfolding light chain antibody domain behaves like an IDP and forms a coacervate that precedes amyloid formation.

After the unfolding of the polypeptide chain, the aggregation is determined by the intrinsic aggregation propensity of the individual segments. We have therefore analyzed the aggregation propensity of FOR-005 using the programs TANGO^38^, Aggrescan^39^, and WALTZ^40^. All programs predict that residues 10–21 and 70–80 have a very high fibril formation propensity in agreement with the cryo-EM and the solid-state NMR data. These regions of the protein must be hot-spots and nucleation points for fibril formation. In Fig. 7D, residues 10–21 are highlighted as aggregation prone region (bold, red line). Other flanking regions which are not predicted to have a high amyloid propensity can potentially become amyloidogenic in the molecular context. e.g. CDR-3 (residues 88–95) is not predicted to be highly amyloidogenic, but packs well against residues 14–22 and 72–85 in the fibril structure.

We hypothesize that charged amino acids in the core of the amyloid fibril structure are the driving force to yield the correct packing arrangement and have to be arranged to yield charge balancing. The disulfide bridge is a strong restraint that determines the arrangement of the flanking segments in the amyloid fibril structure. In FOR005, the disulfide C22-C87 forces the C-terminal part of the protein in close proximity to the amyloidogenic region 1 (10–21). The number of possible topologies is limited. Supplementary Fig. 13 shows potential arrangements and fibril topologies that are obtained after reshuffling of inner and outer segments with respect to the disulfide bond. As a starting point, the cryo-EM topology of FOR005^21^ has been employed. Only the experimental topology yields an effective burial of charged side chains in the core of the amyloid fibril structure. In the native state structure like in the cryo-EM structure, the strands C and F are arranged in an antiparallel fashion. By contrast, strands A and G2 are oriented in parallel in the native state, while they are arranged antiparallel in the fibril structure. Parallel arrangements of the β-strands around the disulfide bond in the fibril structure are not favorable since the long unstructured loop involving residues R49-R60 interferes either with the N- or C-terminus of the protein in each possible topology.

In conclusion, we have structurally characterized early aggregation intermediates in the misfolding of the light chain variable domain of the patient FOR005 implicated in AL-amyloidosis. Unfavorable mutations in the loops CDR-2 and CDR-3 induce local unfolding. Further unfolding is triggered by non-native electrostatic interactions which destabilize the conserved salt bridge network of the native protein. The oligomeric state resembles a phase-separated state which is stabilized by electrostatic interactions between positively and negatively charged residues. The high local protein concentration facilitates interactions between aggregation prone regions of the protein inducing fibril formation. The topology of the fibril state is determined by balanced electrostatic interactions in the core of the amyloid fibril which allows to exclude most other fibril topologies.

## Methods

### Protein expression and purification

Recombinant protein (FOR005 patient, GL and R49G) was produced and purified as described previously^19,20^. Briefly, *E. coli* BL21 with a pET28(b+) vector containing the FOR005 gene was grown in minimal medium. Expression was induced with 1 mM IPTG at OD 0.6–0.8. After an overnight expression at 37 °C, cells were harvested. Subsequently, inclusion bodies were isolated. Protein solubilized from inclusion bodies was subjected to anion exchange chromatography. Refolding of the protein was achieved by dialysis using a 3.5 kDa cut-off membrane and a buffer containing redox agents. Pure protein was obtained after gel filtration chromatography. The molecular mass was confirmed using SDS PAGE and ESI-MS (Supplementary Fig. 5). The total yield was on the order of 25–30 mg protein per liter of M9. To produce isotopically labeled protein, ^15^NH_4_Cl and ^13^C-glucose was employed as nitrogen and carbon sources, respectively. All mutants were expressed and purified using the same protocol. In all biophysical and NMR experiments, the protein was solubilized in a 20 mM phosphate buffer [NaH_2_PO_4_ + Na_2_HPO_4_] adjusted to pH 6.5. The buffer was supplemented with 50 mM NaCl unless specified otherwise.

### SDS PAGE

To confirm the structural integrity of the protein during aggregation, we performed SDS PAGE experiments. Prior to the experiment, protein samples were centrifuged for 3–4 h at 125,000 × *g* to remove pre-formed aggregates using a table-top ultra-centrifuge (Beckman Optima MAX-E equipped with a TLA-45 fixed angle rotor). The samples were incubated afterwards in a shaker (120 rpm, 37 °C) for 8 days. Samples were taken at day 1, 4 and 8. Protein samples were heated to 95 °C for 10 min prior to running the gels.

### ThT experiments

Fibril formation kinetics was measured using a thioflavin T (ThT) assay^41–43^. Prior to the ThT assay, protein solutions were centrifuged for 3–4 h at 125,000 × *g* to remove pre-formed aggregates using a table-top ultra-centrifuge (Beckman Optima MAX-E equipped with a TLA-45 fixed angle rotor). 0.05% sodium azide was added to avoid bacterial growth. As described above, a 20 mM phosphate buffer adjusted to pH 6.5 is employed. Unless specified otherwise, the buffer was supplemented with 50 mM NaCl. Experiments were carried out using a fluorescence spectrometer (FLUOstar Omega, BMG LABTECH), employing a fluorescence excitation and emission wavelength of 448 nm and 482 nm, respectively. The FLUOstar is equipped with 96 well plates (Thermofisher Scientific) accommodating 150 μL in each well. All protein samples were incubated with 10 μM or 25 μM ThT with constant agitation using orbital shaking (1000 rpm). In all experiments, the sample temperature was kept constant at 37 °C. The plates were covered with transparent foil and sealed with lab tape to avoid evaporation. For each protein and concentration, three replicates have been recorded.

The ThT assay shown in Fig. 6A was performed using a GENios plate reader (Tecan Group Ltd., Männedorf, Switzerland) using version XFLUOR4. Fluorescence excitation and emission was set to a wavelength of 440 nm and 480 nm, respectively. All protein samples were incubated in 96 well plates (Thermofisher Scientific) using 25 μM ThT. Samples were constantly agitated at high speed with orbital shaking at 37 °C. Protein samples were filtered using a 0.22 μm membrane before the sample preparation.

To facilitate aggregation, SDS was added in the ThT aggregation assays. The exact amount is stated in the respective figure caption.

### Transmission electron microscopy (TEM)

We performed TEM experiments in order to visualize oligomer and fibril formation. Formvar/Carbon 300 mesh copper coated carbon grids (Electron Microscopy Sciences) were exposed first for 30 s to an argon atmosphere. 5 μL of sample was then added to the grids and incubated for 1 min. Grids were subsequently washed with water and dried using filter paper. For staining, 5–10 μl of uranyl acetate (2%) was added for up to 30 s. Extra stain was removed from the grid using filter paper. Grids were visualized in TEM employing a LIBRA 120 plus (Zeiss) or a JEOL 1400 plus microscope.

### Differential interference contrast (DIC) microscopy

To visualize larger macroscopic structures, we performed DIC microscopy experiments using a DMi8 CS Bino inverted widefield microscope (Leica) using a 100 x (NA = 1.4) oil immersion objective. FOR005, R49G and GL protein solutions were measured daily using a concentration of 50 μM. All samples were centrifuged for 3–4 h at 125,000 × *g* to remove pre-formed aggregates using a table-top ultra-centrifuge (Beckman Optima MAX-E equipped with a TLA-45 fixed angle rotor). 20 μL of sample was pipetted to a channel of an uncoated µ-slide VI 0.4 (ibidi) for each experiment. LAS X (Leica) analysis software and ImageJ was used for image analysis and processing.

### Dynamic light scattering (DLS)

To probe the hydrodynamic radius of the FOR005 oligomers, we performed DLS experiments using a DynaPro NanoStar (Wyatt Technology). All FOR005 variants were measured adjusting the protein concentration to 50 μM. All protein solutions were centrifuged for 3–4 h at 125,000 × *g* to remove pre-formed aggregates using a table-top ultra-centrifuge (Beckman Optima MAX-E equipped with a TLA-45 fixed angle rotor). All experiments were carried out at a temperature of 25 °C in a sample volume of 60 µL employing single use cuvettes. Scattering results were averaged over 5 or 10 measurements, setting the acquisition to 5 s or 10 s with auto-attenuation turned on. The data was analyzed using the software package DYNAMICS V7.

### Circular dichroism (CD) spectroscopy

In order to monitor the secondary structure of the protein during the aggregation process CD spectroscopy experiments were performed using a JASCO J-1500 CD spectrometer. Protein concentrations were 50 μM in all cases. Protein samples were loaded into quartz cells with a path length of 0.1 cm. Spectra were recorded daily. Samples were kept in the quartz cell at all times and incubated in a shaker at 37 °C that was agitated with 120 rpm. CD spectra were measured at 20 °C in the wavelength range from 190 to 260 nm using 0.1 nm intervals, with a response time of 1 s and 5 accumulations. For the analysis of the data, the background buffer signal was subtracted. Data are expressed in terms of mean residue ellipticity *θ*_MRW_ = (deg cm^2^dmol^−1^) × 10^−6^. Prior to the experiments, all protein solutions were centrifuged for 3–4 h at 125,000 × *g* to remove pre-formed aggregates using a table-top ultra-centrifuge (Beckman Optima MAX-E equipped with a TLA-45 fixed angle rotor).

### NMR experiments

All NMR experiments were performed at 25 °C, employing 500 MHz and 600 MHz Bruker Avance III spectrometer equipped with a cryogenic triple resonance probe. The proton chemical shifts were referenced to the water resonance frequency, the ^15^N and ^13^C shifts were referenced indirectly. Assignment of the NMR backbone chemical shifts was achieved using standard triple resonance experiments HNCA, HNCACB and HNCO(CA)CB experiments^44^. HetNOE experiments^45^ were recorded using a protein concentration of 50 μM. hetNOE experiment were conducted in an interleaved fashion with alternating saturated and unsaturated transients. A recycle delay of 3 s was used in successive scans. Peak intensities with and without proton saturation were compared and plotted as a function of residue number. Experiments were carried out at a temperature to 25 °C. NMR data was acquired and processed using Topspin 3.5 (Bruker). CcpNMR-V2 was employed for the sequential assignments and analysis of all NMR experiments^46,47^.

### Quantification of the monomer-dimer equilibrium

To yield concentration dependent chemical shifts, ^1^H,^15^N HSQC experiments have been recorded for the protein concentrations 18 μM, 24 μM, 43 μM, 86 μM, 130 μM, 170 μM, 300 μM, 396 μM, 430 μM, 520 μM, 666 μM, 900 μM, 1100 μM, 1300 μM and 14 μM, 20 μM, 43 μM, 86 μM, 130 μM, 173 μM, 300 μM, 380 μM, 432 μM, 545 μM, 666 μM, 900 μM, 1168 μM for GL and FOR005 respectively, at 25 °C. To calculate *K*_d_, we employed the following equations^48^

1 $${K}_{d}=\frac{{\left[M\right]}^{2}}{\left[D\right]}$$

2 $$x=\left[M\right]+2D$$

3 $$\frac{({CS}-{{CS}}_{{monomer}})}{{{CS}}_{{dimer}}-{{CS}}_{{monomer}}}=\frac{2[D]}{\left[M\right]+2[D]}$$

4 $${CS}=\left({{CS}}_{{dimer}}-{{CS}}_{{monomer}}\right) * \frac{{\left({\left({K}_{d}^{2}+8x{K}_{d}\right)}^{0.5}-{K}_{d}\right)}^{2}}{(8x{K}_{d})}$$

where [*M*], [*D*] reflect the concentration of the monomer and the dimer respectively. *x* represents the total protein concentration and *CS* refers to the chemical shift. Chemical shift changes in ^1^H,^15^N HSQC spectra were calculated using the formula

5 $$\triangle {\delta }_{{NH}}=\sqrt{{\left({\triangle \delta }^{1H}\right)}^{2}+\frac{1}{25}{\left({\triangle \delta }^{15N}\right)}^{2}}$$

### Molecular dynamics simulations

All molecular dynamics (MD) simulations were carried out and analyzed using the Amber18 molecular simulation package^49^. Simulations were performed starting from the FOR005 VL variant for which a crystal structure is available (PDB: 5L6Q) and on in silico generated variants with a R60A substitution as well as the GL sequence (contains S31Y, F48Y, R49G, S51N and A94G substitutions). Each protein was solvated in TIP3P water^50^ in a periodic octahedral box with a minimum distance of protein atoms to the box boundary of 10 Å. The ff14SB force field^51^ was employed and Na^+^ and Cl^-^ ions were added to neutralize the system and reach an ion concentration of 0.15 M. Energy minimization of each system was performed with the sander module of Amber18 (2500 minimization cycles). The systems were heated in steps of 100 K (50 ps per step) to a final temperature of 310 K with the solute non-hydrogen atoms harmonically restraint to the start structure. All bonds involving hydrogen atoms were kept at optimal length. In additional 4 steps the harmonic restraints were removed stepwise. For the subsequent production simulations hydrogen mass repartitioning (HMR) was employed allowing a time step of 4 fs (instead of 2 fs used during heating and equilibration). Unrestrained production simulations were extended to 1.6 µs for each system (with the first 0.4 µs taken as equilibration phase). Coordinates were saved every 4–8 ps. Root mean square deviation (RMSD), root mean square fluctuations (RMSF) and analysis of dihedral angle distributions were performed using the cpptraj module of Amber18.

### Reporting summary

Further information on research design is available in the Nature Portfolio Reporting Summary linked to this article.

## Supplementary information

Supplementary Information

Reporting Summary

## Data Availability

All data generated in this study are provided in the Source Data file [10.6084/m9.figshare.22698466]^52^. Solution-state chemical shift assignments for the native-state of FOR005 is accessible on the BioMagResBank (BMRB) under entry number BMRB 50211 (Solution-state NMR assignments of the patient FOR005 λ-III immunoglobulin light chain variable domain), 51707 (Solution state chemical shift NMR assignment of the patient FOR005 λ-III immunoglobulin light chain variable domain variant FOR005_R49G) and 51708 (Solution state chemical shift NMR assignment of the patient FOR005 λ-III immunoglobulin light chain variable domain germline variant), respectively. In addition, MAS solid-state NMR chemical shift assignments for FOR005 fibrils are accessible on the BioMagResBank (BMRB) under entry number BMRB 50192 (Solid state NMR assignments for the patient FOR005 λ-III immunoglobulin light chain variable domain amyloid fibrils), respectively. The following previously published coordinates were used in Fig. 2: PDB 5L6Q, 2KQN; Fig. 7: 6Z1O. The molecular dynamics trajectories shown in this study will be made available instantly from the corresponding authors upon request. Source data are provided with this paper.

## Source data

Source data
