# Supplementary material for: Mechanistic insights into the aggregation pathway of the patient-derived immunoglobulin light chain variable domain protein FOR005
Source: Nat Commun. 2023 Jun 23;14:3755. doi: 10.1038/s41467-023-39280-0 (PMC10290123; doi:10.1038/s41467-023-39280-0)
Supplement: Supplementary file 1 — Supplementary Information [file 41467_2023_39280_MOESM1_ESM.pdf]

Supplementary Information

for the manuscript

Mechanistic insights into the aggregation pathway of the patient-derived immunoglobulin light chain protein FOR005

Tejaswini Pradhan,[a,b] Riddhiman Sarkar,[a,b] Kevin M. Meighen-Berger,[c] Matthias J. Feige,[c] Martin Zacharias,[d] and Bernd Reif [a,b]*

[a] Bavarian NMR Center (BNMRZ), Department of Bioscience, TUM School of Natural Sciences, Technical University Munich, Lichtenbergstr. 4, 85747 Garching, Germany

[b] Institute of Structural Biology (STB), Helmholtz-Zentrum München (HMGU), Ingolstädter Landstr. 1, 85764 Neuherberg, Germany

[c] Center for Functional Protein Assemblies (CPA), Department of Bioscience, TUM School of Natural Sciences, Technical University Munich, Ernst-Otto-Fischer-Straße 8, 85748 Garching, Germany

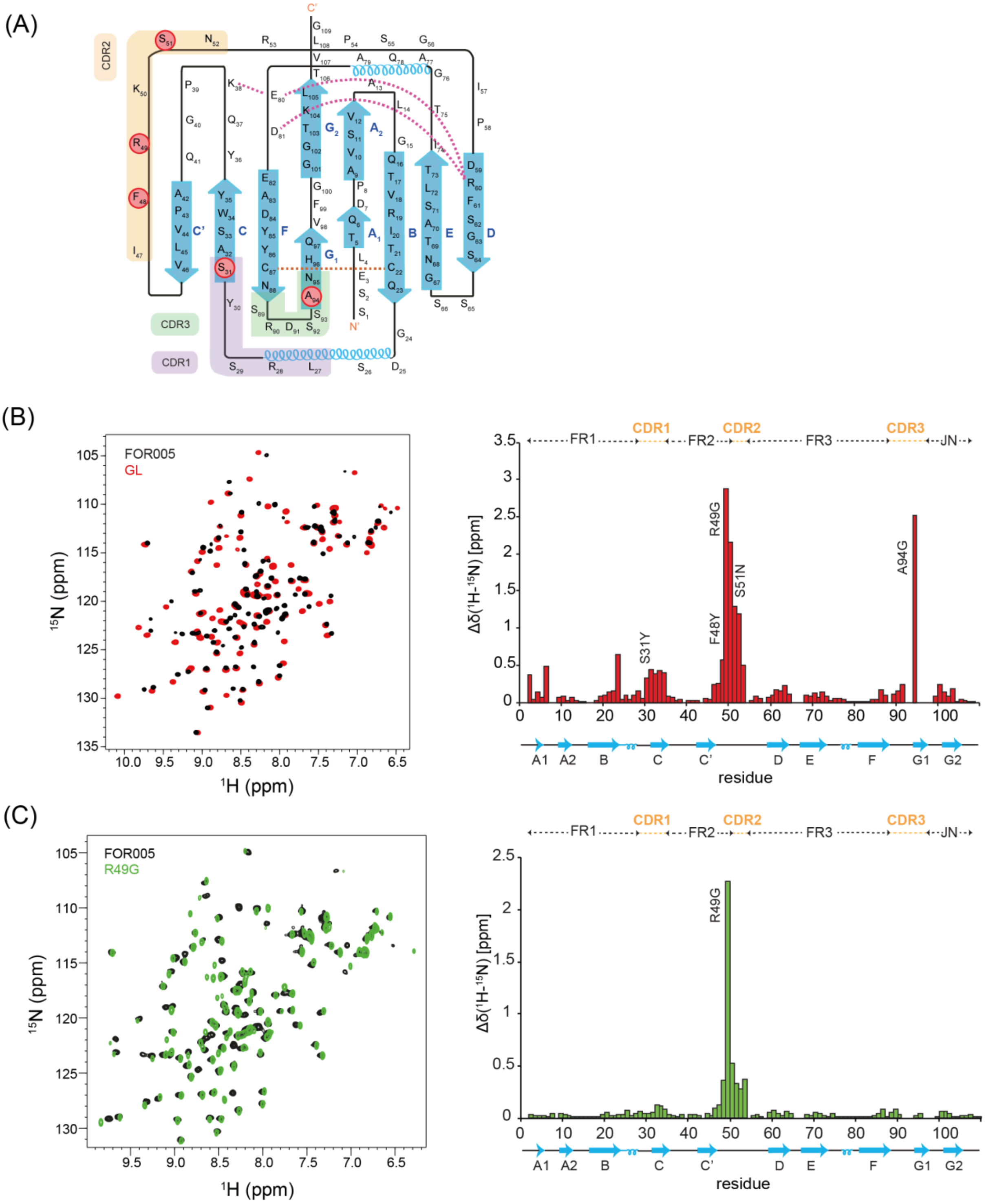

Supplementary Figure 1. *(A) Topology of the native state V_L domain. β-sheets are shaded in blue, the Complementarity Determining Regions (CDRs) are drawn in purple, yellow and green, respectively. Conserved salt bridges and the disulfide bond are indicated with dashed lines. (B) ^{1}H,^{15}N HSQC of FOR005 (black) superimposed with a spectrum of GL in red. Chemical shift perturbations of the FOR005 patient protein in comparison to GL are shown in the panel on the right. (C) ^{1}H,^{15}N HSQC of FOR005 (black) superimposed with a spectrum of R49G in green. Chemical shift perturbations of the FOR005 patient protein in comparison to the single point mutant R49G are represented in the panel on the right. Mutations from patient to germline (and patient to R49G) are indicated in the chemical shift diagrams.*

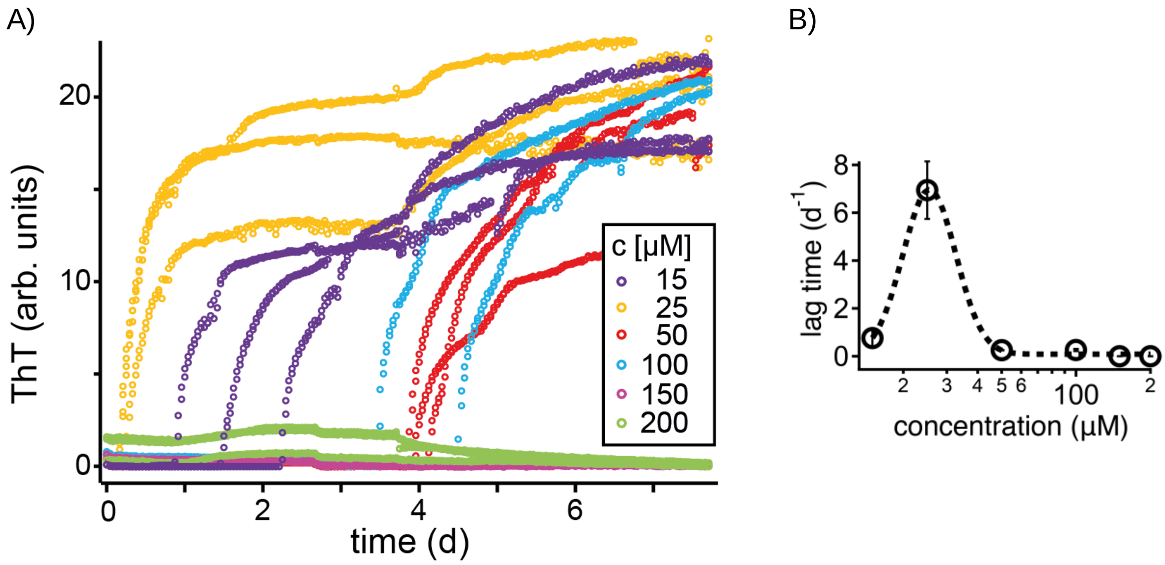

Supplementary Figure 2. *ThT aggregation kinetics. (A) Concentration dependence of the aggregation kinetics of the patient protein FOR005. For each concentration, three replicates are recorded. 500 µM SDS was added to all protein solutions to facilitate aggregation. n=3 independent ThT experiments for protein concentrations of 15 (purple), 25 (yellow), 50 (red), 100 (blue), 150 (maroon) and 200 µM (green) have been recorded. (B) Inverse lag time as a function of the FOR005 protein concentration. The individual ThT data curves are shown in panel (A). We find that high protein concentrations which favor the dimeric state are protective against aggregation. Also, aggregation is slow at very low concentrations which impede primary nucleation. The fastest aggregation is observed for an intermediate concentration of 25 µM. These results are consistent with previous studies by Fink, Ramirez-Alvarado and coworkers in which the concentration dependence of protein aggregation in AL amyloidosis has been systematically studied[1,2]. For protein concentrations of 15, 25, 50, 100, 150 and 200 µM, the fit was yielding inverse lag times of (0.76±0.35)d^{-1}, (6.94±1.20)d^{-1}, (0.25±0.01)d^{-1}, (0.26±0.05)d^{-1}, ∞, and ∞ respectively. Source data are provided as a Source Data file.*

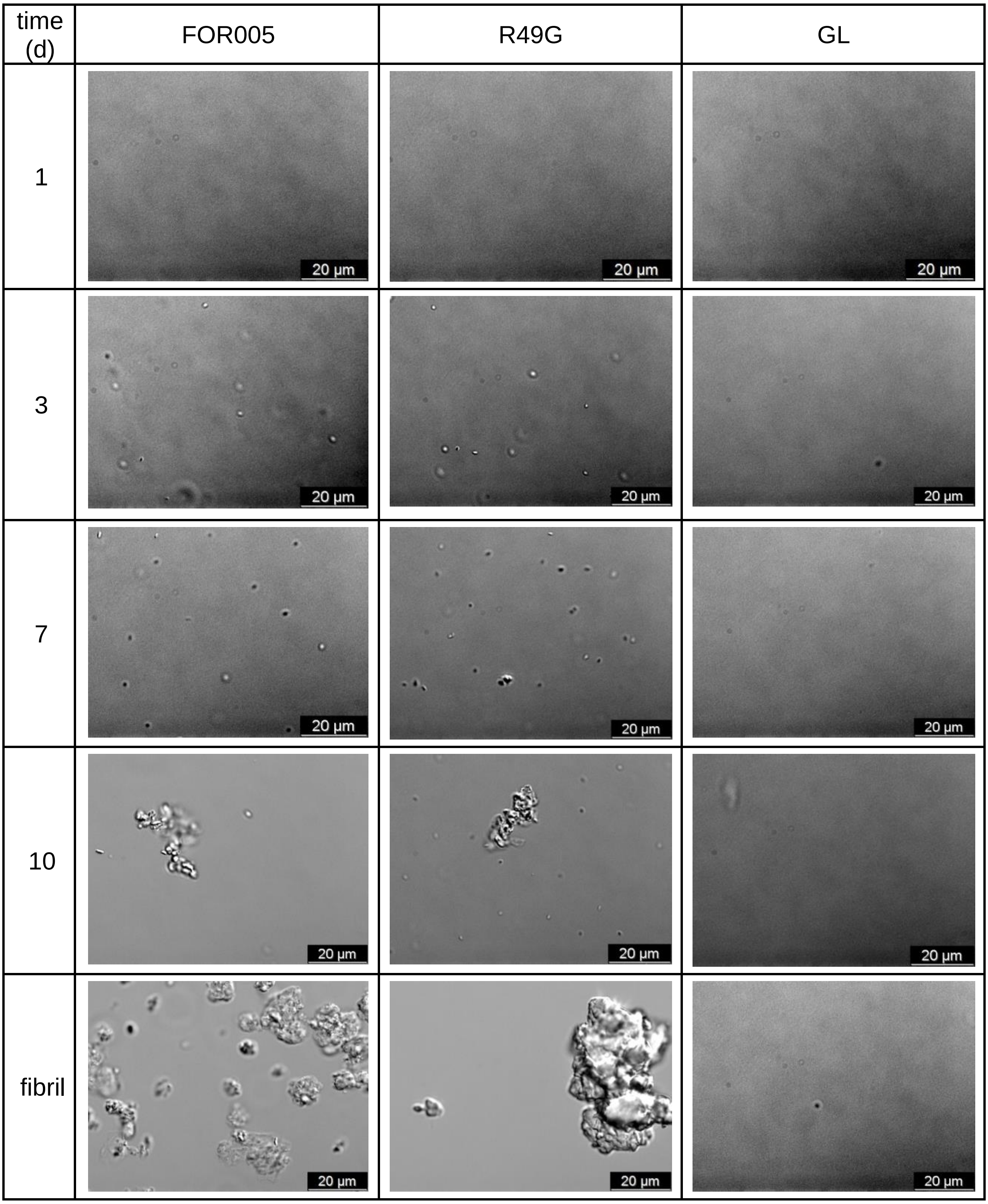

Supplementary Figure 3. *Representative Differential Interference Contrast (DIC) microscopy images of the patient protein FOR005, R49G and the germline protein GL as a function of time. The protein solution was ultracentrifuged for 3-4 hours prior to incubation (Optima MAX-E ultracentrifuge, Beckman, 125.000g). After 3 days, the FOR005 and R49G protein solutions show spherical particles that have a diameter of approximately 0.5 µm. These oligomeric aggregates are not observable in the GL protein solution. Full images are provided in the Source Data file.*

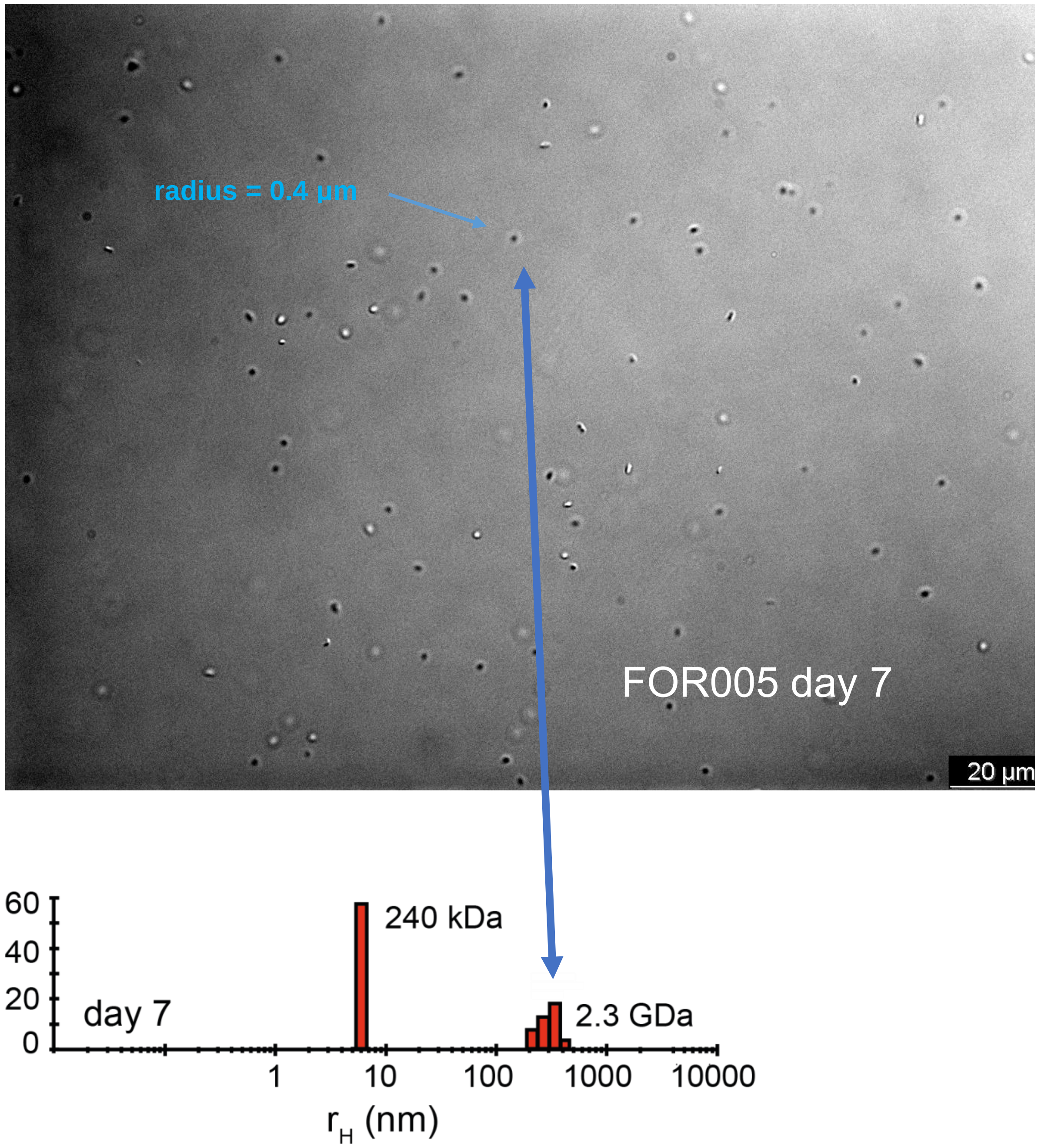

Supplementary Figure 4. *Comparison of DIC and DLS data for the patient protein FOR005 after an incubation time of 7 days. The GDa complexes observed in the DLS experiments match in size the high molecular weight particles observed in the DIC experiments. Image analysis of n=30 DIC particles using ImageJ and LAS X (Leica) analysis software yields an average radius of (0.4±0.2) µm. Source data are provided as a Source Data file.*

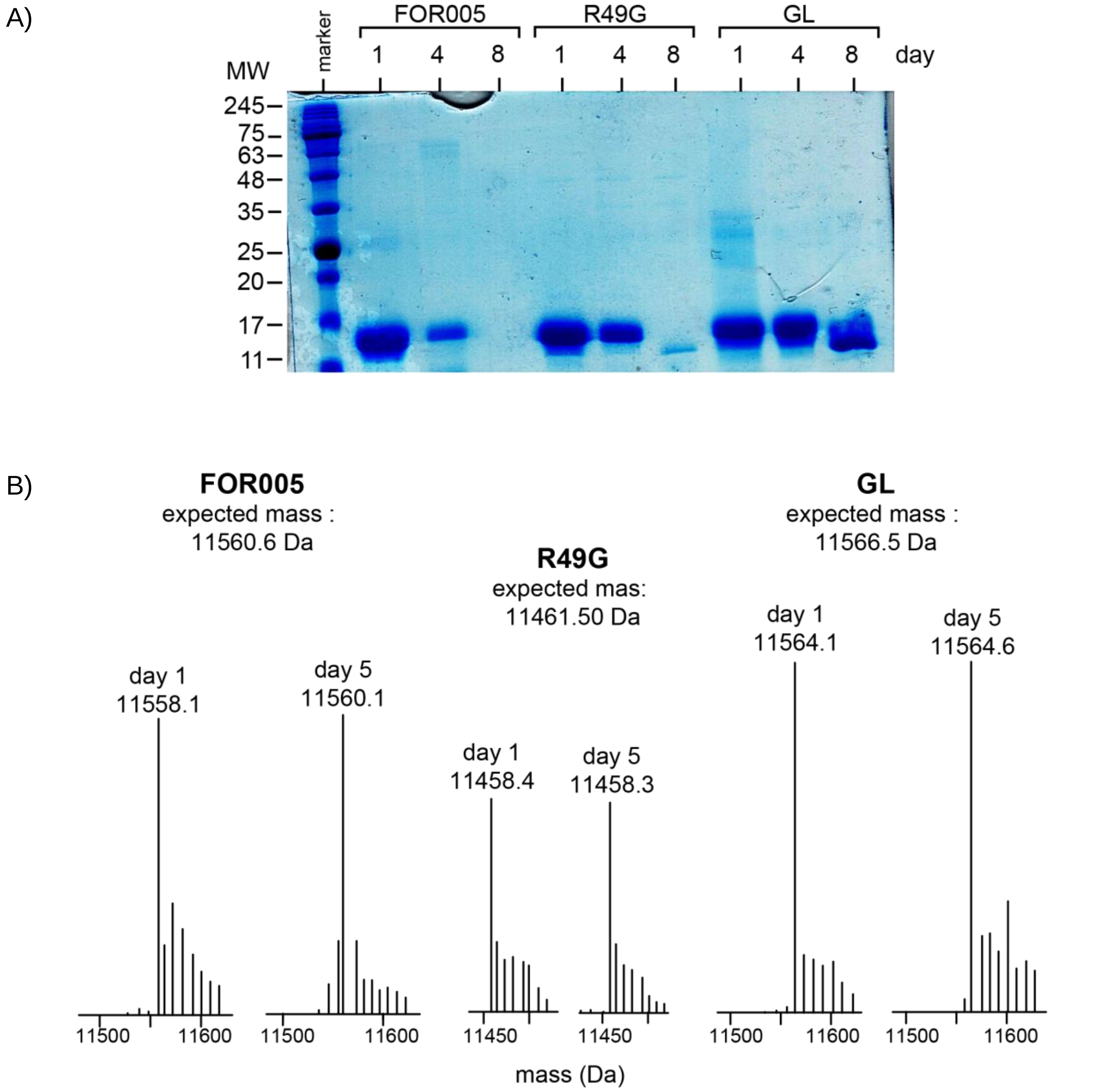

Supplementary Figure 5. *The chemical integrity of all proteins is preserved during aggregation. (A) Aggregation kinetics of FOR005 and GL followed by SDS PAGE. For the germline protein, the monomer band is observed at all times. By contrast, the monomer band disappears for FOR005 and R49G after day 4. Instead, multiple faint bands appear at high molecular weight, indicating that these proteins form SDS stable oligomers. For all three proteins, no degradation bands at lower molecular weights are observed. All PAGE experiments were carried out using a protein concentration of 50 µM (data representative from two independent assays). B) ESI-MS analysis of the patient protein FOR005, the single point mutant R49G and the germline protein GL. Sample have been taken at the beginning of the aggregation kinetics and 4 days later. All proteins retain their structural integrity in the time course of the experiment. Source data are provided as a Source Data file.*

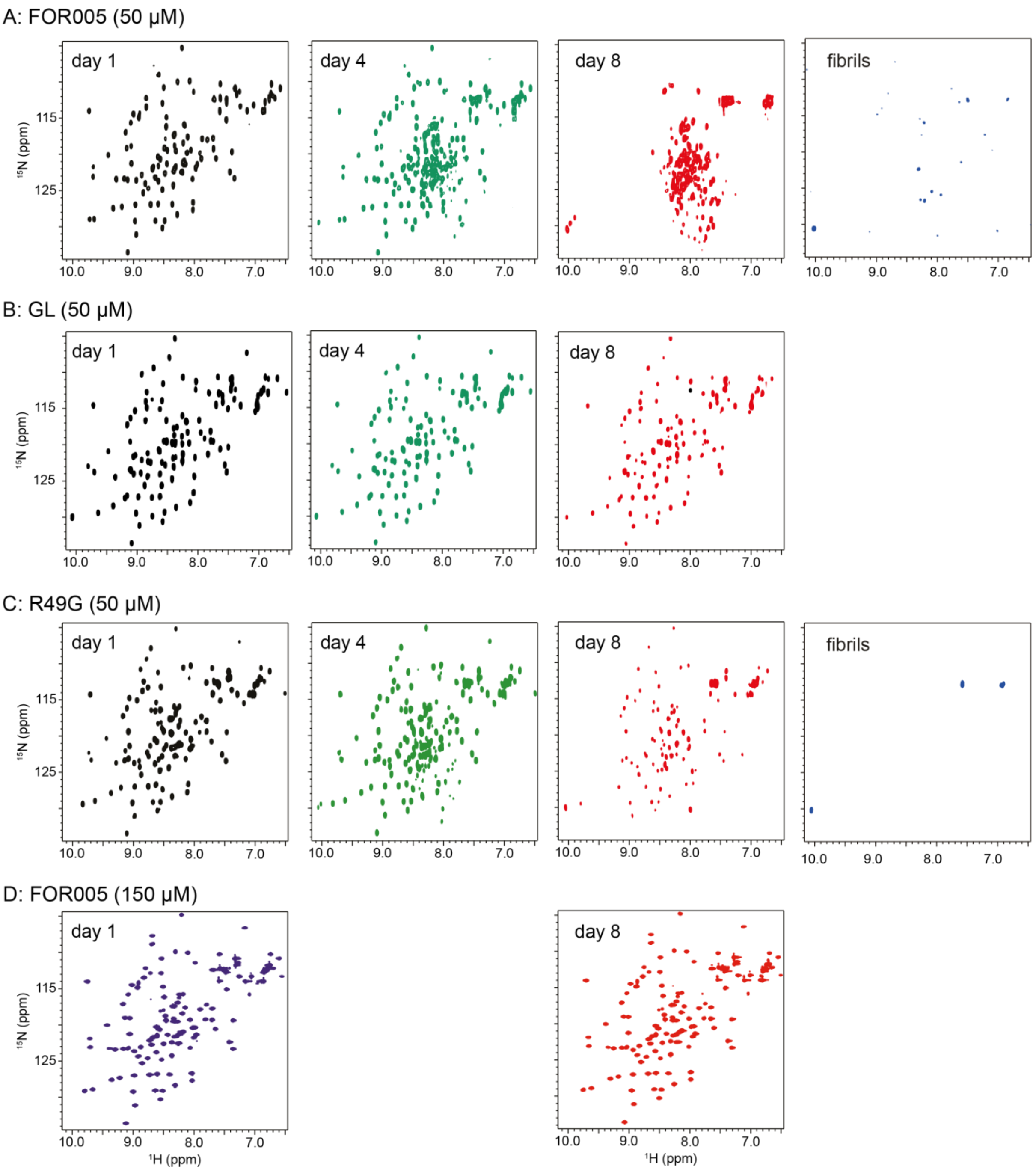

Supplementary Figure 6. *Aggregation kinetics of different FOR005 variants followed by solution state NMR HSQCs. The ^{1}H,^{15}N HSQC spectra were acquired over a period of 1 week for a 50 µM sample of FOR005 (A), GL (B) and R49G (C). After an incubation time of 30 days, the spectrum "fibril" was recorded. All experiments were recorded at a temperature of 25°C. For FOR005, new peaks become visible after a period of 3 days which indicate protein unfolding. For GL, no additional cross peaks appear in the spectra over time. For R49G, a few new peaks are observed in the course of the experiment. (D) ^{1}H,^{15}N HSQC spectra for FOR005 at a protein concentration of 150 µM recorded at day 1 (blue) and day 8 (red). Under these conditions, no aggregation is observed. All experiments were recorded at a temperature of 25°C.*

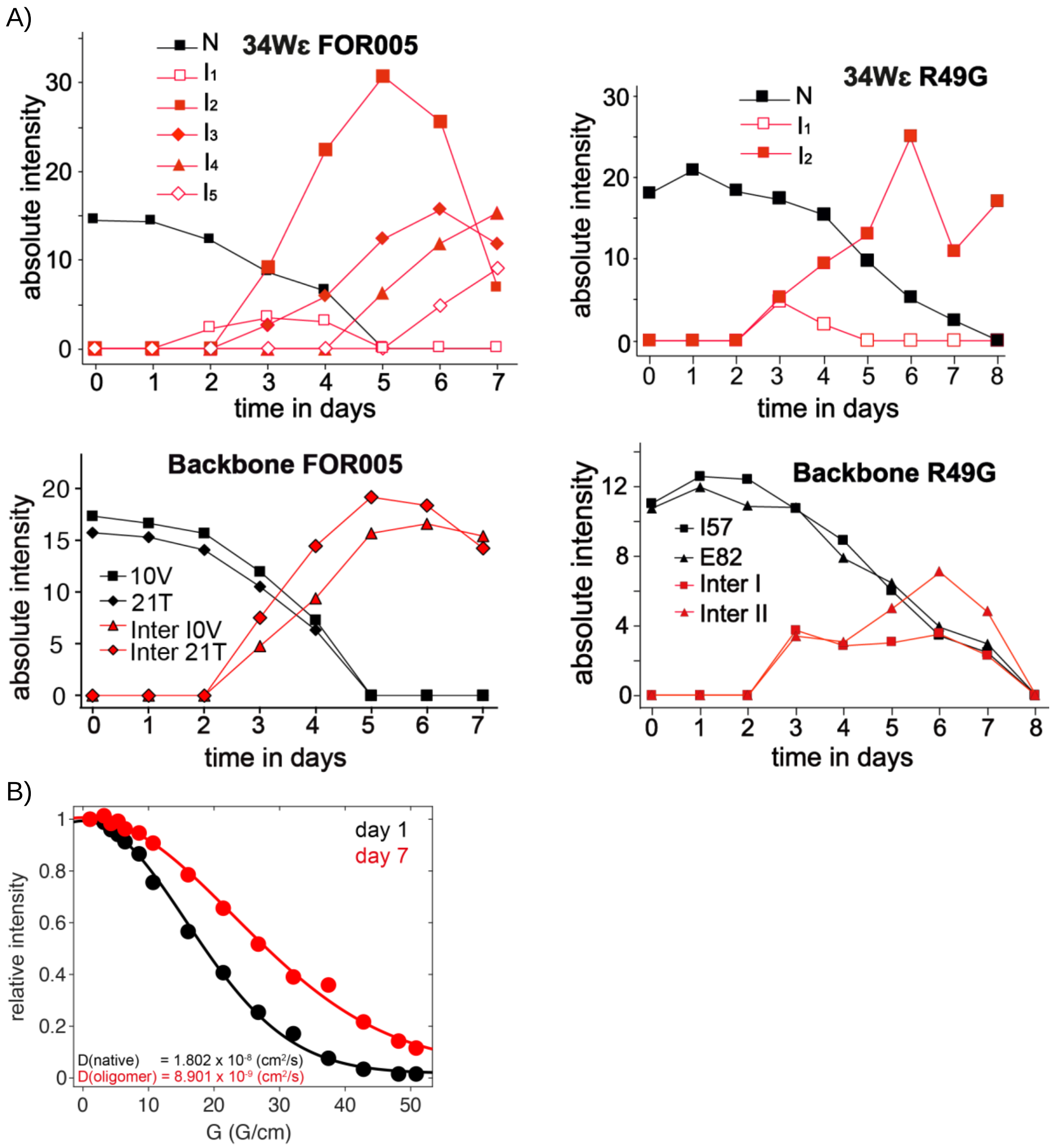

Supplementary Figure 7. *(A) W34ε cross peak intensity as a function of time for FOR005 and R49G (top). Backbone cross peak intensities as a function of time for FOR005 and R49G (bottom). Intensity values were extracted from the 50 μM protein preparations shown in Fig. S6. For FOR005 intermediate state, backbone intensities for the assigned residues V10 and T21 are shown. For R49G, two arbitrary backbone resonances have been selected. (B) Diffusion Ordered Spectroscopy (DOSY) NMR experiments for the patient protein FOR005 at day 1 and day 7 of the aggregation kinetics. The diffusion constant decreases in the course of time indicating an increase of the molecular weight of the complex. Source data are provided as a Source Data file.*

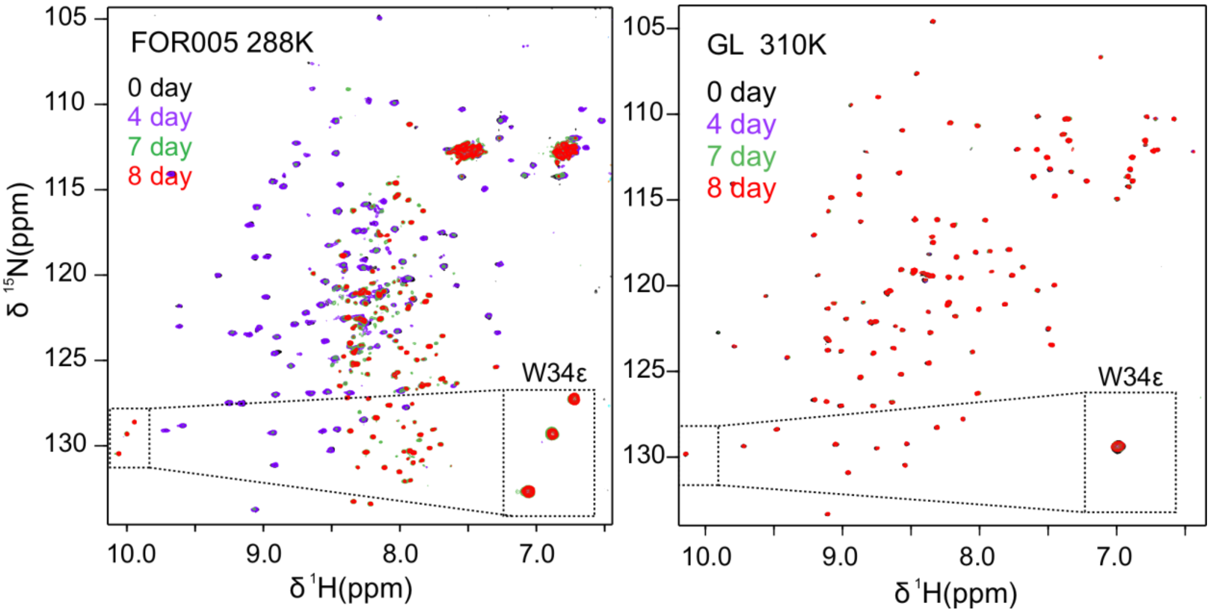

Supplementary Figure 8. *Superposition of the ^{1}H,^{15}N HSQC spectra obtained for FOR005 and GL recorded at 15°C and 37°C, respectively, as a function of time. A protein concentration of 50 µM was employed in both experiments. FOR005 and GL unfold at a temperature of (43.5±0.1)°C and (56.3±0.1)°C[3]. GL is stable over a period of 1 week at a temperature of 19°C below the thermal unfolding transition. By contrast, the patient protein FOR005 is unstable even at temperatures 28 °C below the thermal unfolding temperature. Thermodynamic destabilization seems therefore not sufficient to explain unfolding and fibril formation of FOR005.*

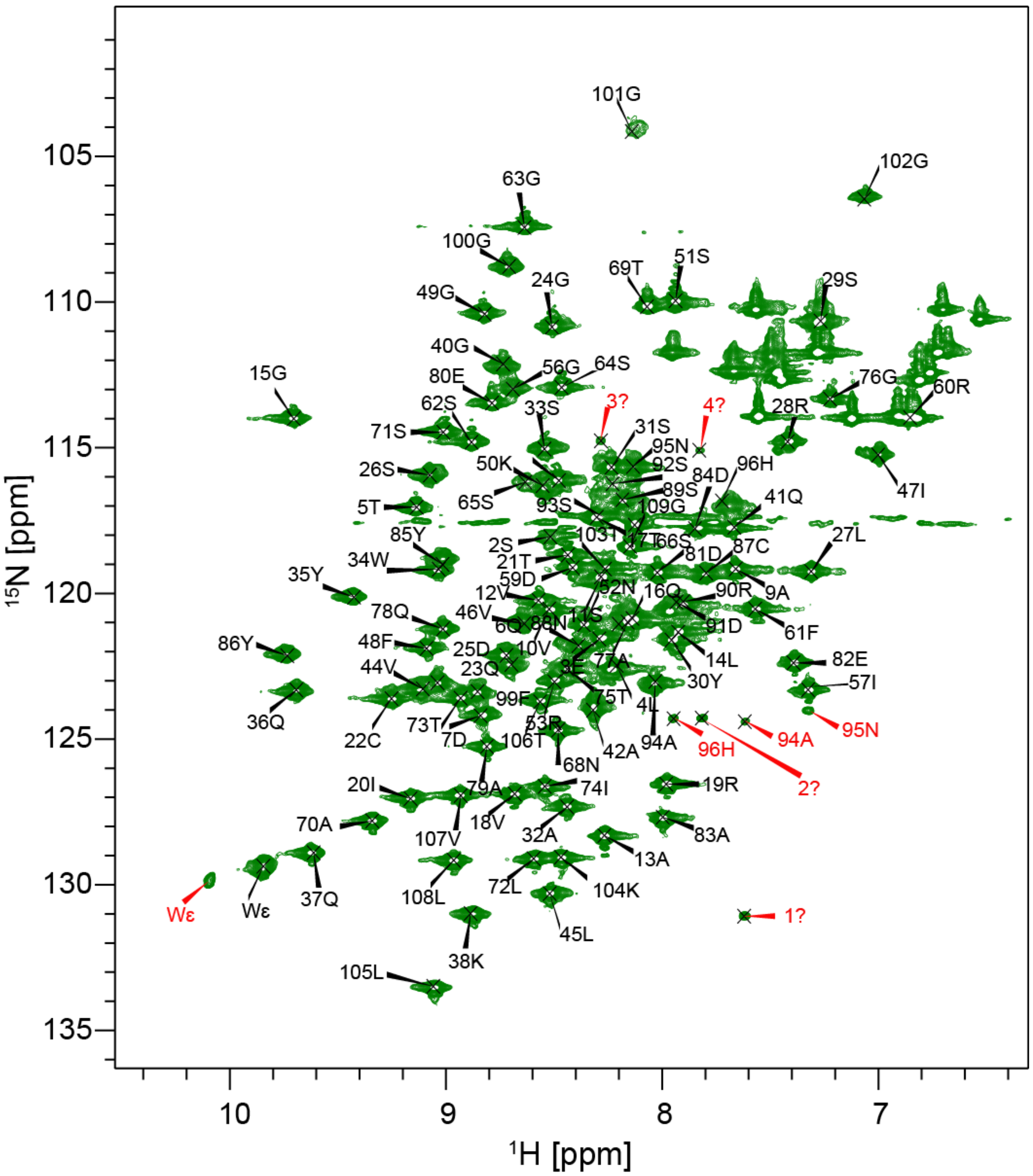

Supplementary Figure 9. *^{1}H,^{15}N HSQC spectrum obtained for FOR005-R49G obtained after several weeks. A protein concentration of 700 μM was employed in the experiment. The peaks assigned in black are from the natively folded protein, peaks assigned in red are from the intermediate state that is partially unfolded. The spectrum was recorded at a temperature of 25°C.*

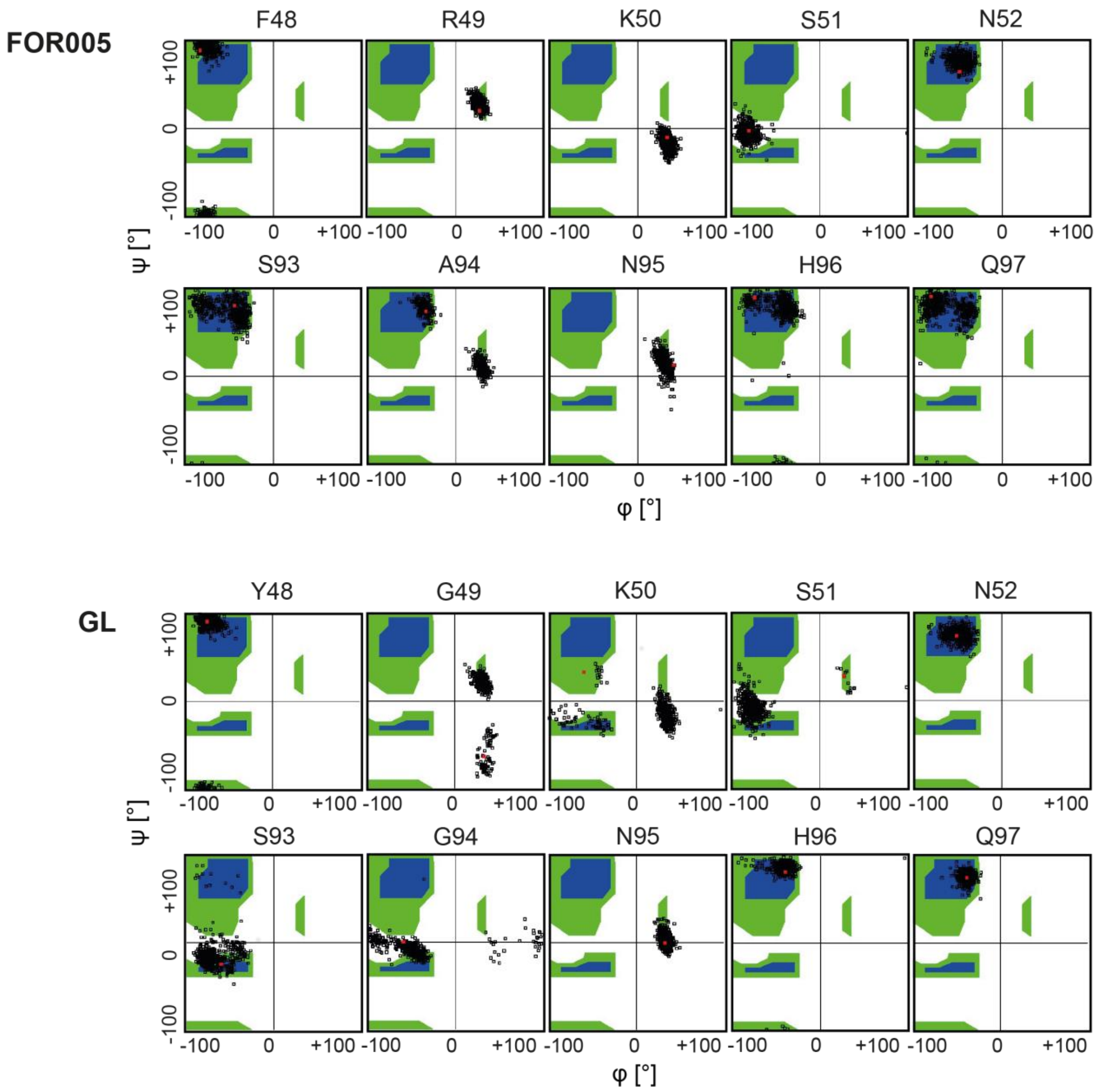

Supplementary Figure 10. *Distribution of backbone dihedral angles phi and psi during the MD simulation of the patient protein FOR005 (top) and GL (bottom). The sampled backbone dihedral angles are represented in a Ramachandran diagram for residues 48 to 52, and 93 to 97 (black open squares). Favorable regions are indicated in blue, the extended allowed region in green. Note, that R49 and A94 in FOR005 adopt partially unfavorable backbone conformations in Ramachandran space, resulting in an energetic penalty for forming the loop structure. For G49 and G94 in GL, similar states are tolerated and not associated with an energetic penalty. With the exception of S93 which flips from one favorable to another favorable region in the Ramachandran diagram, similar backbone conformations are samples during the trajectory for both FOR005 and GL.*

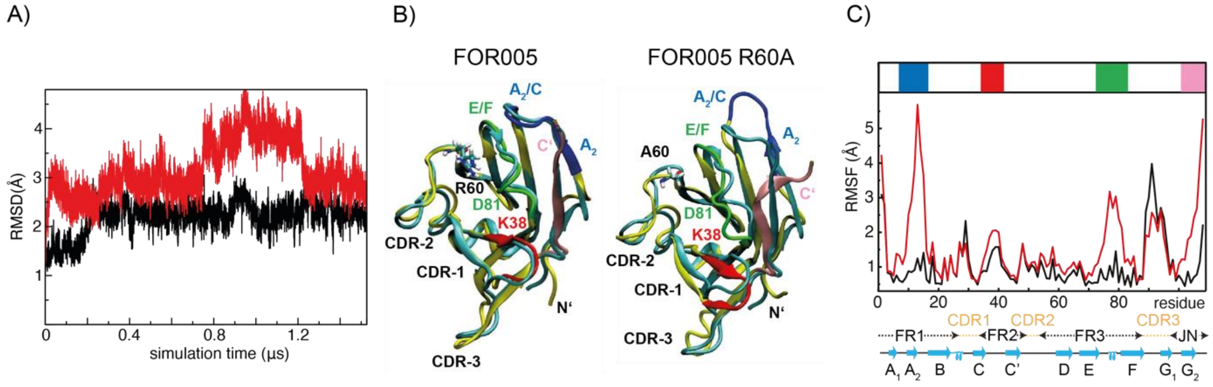

Supplementary Figure 11. *Molecular dynamics simulations of the patient protein FOR005 and the single point mutant R60A. (A) The simulation was carried out in explicit solvent for a duration of 1.6 μs. Black and red curves refer to the patient protein FOR005 and FOR005-R60A, respectively. (B) Superposition of the backbone structures for FOR005 and FOR005 R60A for a snapshot in the simulation at t=0 and t=1 ns. (C) Root-mean-square fluctuations (RMSF) as a function of residue calculated from the MD trajectory (1.6 μs, at 310 K) for the patient sequence FOR005 (black) and the single point mutant FOR005-R60A (red). The color code on top of the figure highlights regions in the protein that become dynamic as a consequence of the point mutation R60A and match the color of particular secondary structure elements in (B).*

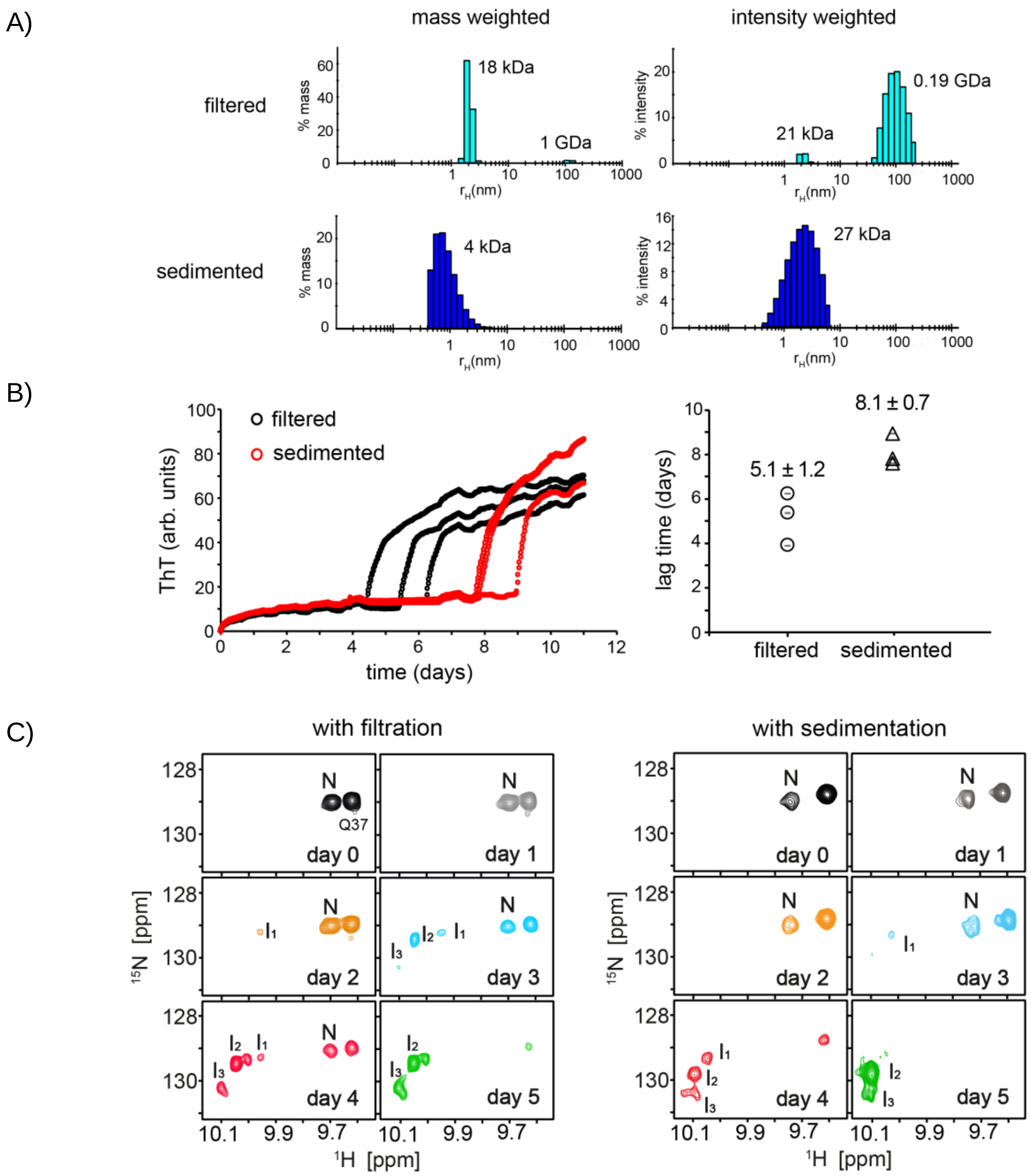

Supplementary Figure 12. *Impact of sample preparation on the removal of high-molecular weight aggregates and the aggregation kinetics of the patient protein FOR005. (A) DLS size distribution for a freshly prepared 50 µM FOR005 protein solution that is filtered using a 0.22 µm cut-off membrane (top), and that is sedimented using an ultra-centrifuge (Optima MAX-E ultracentrifuge, Beckman, 4h, 125.000g) (bottom). Only the intensity weighted analysis shows that high molecular weight aggregates are present in the sample. These aggregates contribute less than 1 % to the total sample (mass weighted analysis). (B) ThT aggregation assay of a 50 µM FOR005 protein solution that was treated as described above. Black and red circles represent the filtered and the sedimented protein solution, respectively. n=3 independent ThT experiments for filtered and sedimented FOR005 protein solutions have been recorded. Sedimentation increases the aggregation lag time from T= (5.1±1.2)d to (8.1±0.7)d (right). (C) NMR aggregation assay. As described above, the 50 µM FOR005 protein solution was either filtered (left) or sedimented (right) prior to the experiment. We find that high molecular weight particles that are present to small amounts at the beginning of the experiment do not impact the aggregation kinetics. At the same time, the appearance of new resonances is not affected. All experiments were recorded at a temperature of 25°C using the same sample conditions. Source data are provided as a Source Data file.*

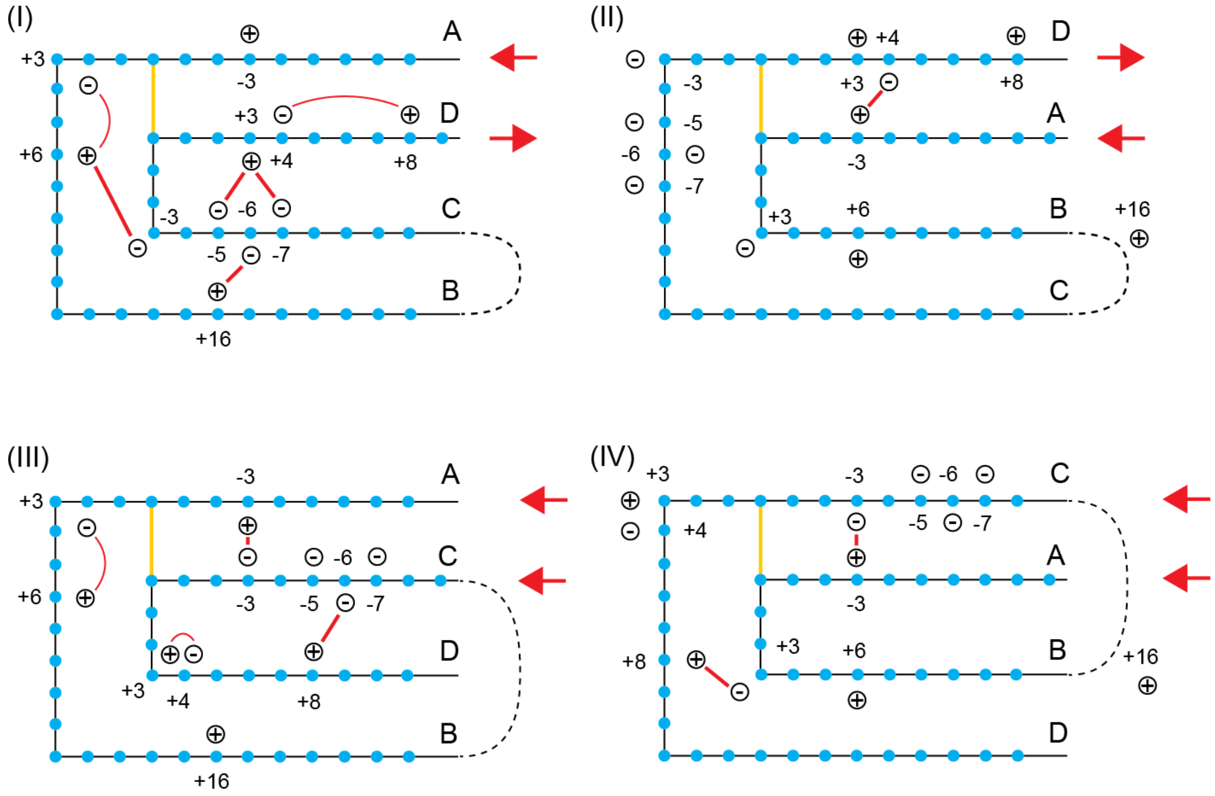

Supplementary Figure 13. *FOR005 fibril topologies after reshuffling of strands around the most amyloidogenic strand A. Strands are named A, B, C, D according to their position with respect to the disulfide bond. In total, there are 4 possible arrangements. Strands A and B can be either positioned on the surface, while strands C and D are located in the core, or the reverse. In addition, the two inner strands can be reshuffled. (I) Experimental topology in the cryo-EM structure of FOR005[4]. The position of every charged amino acid is indicated relative to the disulfide. In this topology, every charged side chain is compensated with an opposite charged residue in the core of the amyloid fibril structure. Strands A/B and C/D are arranged in an antiparallel fashion. (II) Topology after exchange of the outer (A/B) and inner strands (C/D) (in comparison to topology I). A shift of the disulfide does not yield a charge compensation and retains a frustrated structure. This topology is thus not favorable. (III) Topology after reshuffling of the two inner strands C and D (in comparison to topology I). Strands A/B and C/D are arranged in parallel. In parallel strands, a shift of the disulfide does not result in a different arrangement of the charged side chains. The loop connecting strands B and C interferes with the C-terminus and thus yields a non-favorable topology. (IV) Topology after reshuffling of the outer strands C and D (with respect to topology II). Strands A/B and C/D are arranged in parallel. The loop connecting strands B and C interferes with the N-terminus and thus yields a non-favorable topology.*

Supplementary References

1. Qin, Z. J., Hu, D. M., Zhu, M. & Fink, A. L. Structural characterization of the partially folded intermediates of an immunoglobulin light chain leading to amyloid fibrillation and amorphous aggregation. Biochemistry 46, 3521-3531 (2007).
2. Blancas-Mejia, L. M., Misra, P. & Ramirez-Alvarado, M. Differences in Protein Concentration Dependence for Nucleation and Elongation in Light Chain Amyloid Formation. Biochemistry 56, 757-766 (2017).
3. Rottenaicher, G. J., Weber, B., Rührnößl, F., Kazman, P., Absmeier, R. M., Hitzenberger, M., Zacharias, M. & Buchner, J. Molecular mechanism of amyloidogenic mutations in hypervariable regions of antibody light chains. J. Biol. Chem. 296, e100334 (2021).
4. Radamaker, L., Baur, J., Huhn, S., Haupt, C., Hegenbart, U., Schönland, S., Bansal, A., Schmidt, M. & Fändrich, M. Cryo-EM reveals structural breaks in a patient-derived amyloid fibril from systemic AL amyloidosis. Nat. Commun. 12, e875 (2021).
